# Local tumor control and neurological outcomes after surgery for spinal hemangioblastomas in sporadic and von Hippel–Lindau disease: A multicenter study

**DOI:** 10.1093/neuonc/noaf041

**Published:** 2025-02-15

**Authors:** Johannes Wach, Alim Emre Basaran, Martin Vychopen, Tarik Tihan, Maria Wostrack, Vicki M Butenschoen, Bernhard Meyer, Sebastian Siller, Nils Ole Schmidt, Julia Onken, Peter Vajkoczy, Alejandro N Santos, Laurèl Rauschenbach, Philipp Dammann, Ulrich Sure, Jan-Helge Klingler, Roberto Doria-Medina, Jürgen Beck, Bianca-Ioana Blaß, Christine Julia Gizaw, Romina Hohenhaus, Sandro Krieg, Obada T Alhalabi, Lukas Klein, Claudius Thomé, Nikolaus Kögl, Przemyslaw Kunert, Tomasz Czernicki, Tobias Pantel, Maximilian Middelkamp, Sven Oliver Eicker, Ahed H Kattaa, David J Park, Steven D Chang, Fatma Kilinc, Marcus Czabanka, Erdem Güresir

**Affiliations:** Comprehensive Cancer Center Central Germany, Partner Site Leipzig, Leipzig, Germany; Department of Neurosurgery, University Hospital Leipzig, Leipzig, Germany; Comprehensive Cancer Center Central Germany, Partner Site Leipzig, Leipzig, Germany; Department of Neurosurgery, University Hospital Leipzig, Leipzig, Germany; Comprehensive Cancer Center Central Germany, Partner Site Leipzig, Leipzig, Germany; Department of Neurosurgery, University Hospital Leipzig, Leipzig, Germany; Department of Pathology, Miller School of Medicine, University of Miami, Miami, Florida, USA; Division of Neuropathology, UCSF School of Medicine, San Francisco, California, USA; Department of Neurosurgery, Technical University of Munich, School of Medicine, Klinikum rechts der Isar, Germany; Department of Neurosurgery, Technical University of Munich, School of Medicine, Klinikum rechts der Isar, Germany; Department of Neurosurgery, Technical University of Munich, School of Medicine, Klinikum rechts der Isar, Germany; Department of Neurosurgery, University Hospital, University of Regensburg, Regensburg, Germany; Department of Neurosurgery, University Hospital, University of Regensburg, Regensburg, Germany; Department of Neurosurgery, Charité—Universitätsmedizin Berlin, Corporate Member of Freie Universität Berlin and Humboldt-Universität zu Berlin, Berlin, Germany; Department of Neurosurgery, Charité—Universitätsmedizin Berlin, Corporate Member of Freie Universität Berlin and Humboldt-Universität zu Berlin, Berlin, Germany; Department of Neurosurgery, Royal Adelaide Hospital, Adelaide, Australia; Department of Neurosurgery and Spine Surgery, University Hospital Essen, Essen, Germany; DKFZ-Division Translational Neurooncology at the WTZ, DKTK Partner Site, University Hospital Essen, Essen, Germany; Department of Neurosurgery and Spine Surgery, University Hospital Essen, Essen, Germany; Department of Neurosurgery and Spine Surgery, University Hospital Essen, Essen, Germany; Department of Neurosurgery and Spine Surgery, University Hospital Essen, Essen, Germany; Department of Neurosurgery, Medical Center—University of Freiburg, Freiburg, Germany; Department of Neurosurgery, Medical Center—University of Freiburg, Freiburg, Germany; Department of Neurosurgery, Medical Center—University of Freiburg, Freiburg, Germany; Department of Neurosurgery, Medical Center—University of Freiburg, Freiburg, Germany; Department of Neurosurgery, Medical Center—University of Freiburg, Freiburg, Germany; Department of Neurosurgery, Medical Center—University of Freiburg, Freiburg, Germany; Medical Faculty, Heidelberg University, Heidelberg, Germany; Department of Neurosurgery, Heidelberg University Hospital, Heidelberg, Germany; Medical Faculty, Heidelberg University, Heidelberg, Germany; Department of Neurosurgery, Heidelberg University Hospital, Heidelberg, Germany; Medical Faculty, Heidelberg University, Heidelberg, Germany; Department of Neurosurgery, Heidelberg University Hospital, Heidelberg, Germany; Department of Neurosurgery, Medical University Innsbruck, Innsbruck, Austria; Department of Neurosurgery, Medical University Innsbruck, Innsbruck, Austria; Department of Neurosurgery, Medical University of Warsaw, Warsaw, Poland; Department of Neurosurgery, Medical University of Warsaw, Warsaw, Poland; Department of Neurosurgery, University Medical Center Hamburg-Eppendorf, Hamburg, Germany; Department of Neurosurgery, University Medical Center Hamburg-Eppendorf, Hamburg, Germany; Department of Spine and Scoliosis Surgery, Lubinus Clinicum, Kiel, Germany; Department of Neurosurgery, University Medical Center Hamburg-Eppendorf, Hamburg, Germany; Department of Neurosurgery, Stanford University School of Medicine, Stanford, California, USA; Department of Neurosurgery, Stanford University School of Medicine, Stanford, California, USA; Department of Neurosurgery, Stanford University School of Medicine, Stanford, California, USA; Department of Neurosurgery, University Hospital Frankfurt, Frankfurt am Main, Germany; Department of Neurosurgery, University Hospital Frankfurt, Frankfurt am Main, Germany; Comprehensive Cancer Center Central Germany, Partner Site Leipzig, Leipzig, Germany; Department of Neurosurgery, University Hospital Leipzig, Leipzig, Germany

**Keywords:** complete resection, multicenter study, neurological outcomes, progression-free survival, spinal hemangioblastomas, von Hippel–Lindau disease

## Abstract

**Background:**

Spinal hemangioblastomas (sHBs) are rare vascular tumors with significant neurological implications. Their management, particularly in von Hippel–Lindau (VHL) disease, remains challenging due to recurrence and functional decline. Timely identification and intervention are critical for optimal outcomes.

**Methods:**

This international, multicenter retrospective cohort study included 357 patients (199 VHL-associated, 158 sporadic) from 13 neuro-oncological centers. Clinical and imaging data were analyzed to assess progression-free survival (PFS) and functional outcomes using the modified McCormick Scale (mMCS) at 12 months. Secondary analyses identified factors associated with VHL disease in sHBs.

**Results:**

Complete resection was achieved in 87.7% of cases, leading to significantly improved PFS at 72 months (sporadic: 95.1%, VHL-associated: 91.1%; hazard ratio: 0.18, 95% CI: 0.08–0.4). Multivariable analysis identified predictors of unfavorable outcomes at 12 months: preoperative mMCS ≥2 (odds ratio [OR]: 5.17, *P* = .008), intramedullary tumor location (OR: 9.48, *P* = .01), and preoperative bleeding (OR: 31.12, *P* = .02). Factors independently associated with VHL disease in sHBs included non-cervical tumor location (OR: 2.08, *P* = .004), intramedullary growth (OR: 2.39, *P* < .001), and age <43 years (OR: 3.24, *P* < .001). Functional improvements were observed in most patients, particularly those with sporadic sHBs.

**Conclusions:**

Complete surgical resection is essential for long-term tumor control and favorable functional outcomes in both sporadic and VHL-associated sHBs. Early intervention, particularly in mild symptomatic and progressive cases, before neurological deterioration or hemorrhage, optimizes recovery. This study, the largest of its kind in a multicentric international setting, provides robust evidence to guide the management of both sporadic and VHL-associated sHBs.

## Graphical Abstract

**Graphical Abstract:**
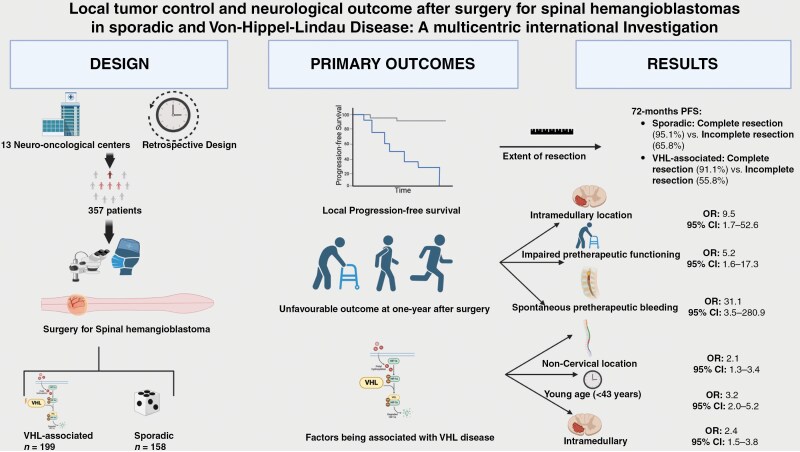

**Key Points**

Significance of complete resection (CR): CR is associated with significantly improved progression-free survival (PFS) in patients with spinal hemangioblastomas (sHBs), with a 72-month PFS rate of 95.1% in sporadic cases and 91.1% in von Hippel–Lindau (VHL)-associated cases.
Neurological outcomes and predictors: Impaired preoperative function, intramedullary tumor location, and preoperative bleeding are significant predictors of unfavorable outcomes at 12 months post-surgery.
Factors associated with VHL disease: Non-cervical tumor location, intramedullary growth, and younger age (<43 years) are strongly linked to VHL-associated sHBs, aiding in early diagnostic strategies.
Clinical impact: Early surgical intervention with CR in symptomatic and progressive sHBs is critical for optimizing long-term tumor control and neurological recovery.

**Importance of the Study**

This study represents the largest international multicenter investigation on spinal hemangioblastomas (sHBs), providing robust evidence for the critical role of complete resection in long-term tumor control and functional outcomes. By analyzing 357 patients from 13 neuro-oncological centers, the findings identify key prognostic factors and clinical differences between sporadic and von Hippel–Lindau (VHL)-associated sHBs. This work underscores the importance of early diagnosis and tailored surgical strategies, particularly in high-risk VHL patients, to improve clinical outcomes and guide future management practices.

Spinal hemangioblastomas (sHBs) are rare, benign vascular tumors, primarily intramedullary, that arise within the spinal cord.^1^ They occur either sporadically or in 25%-33% of cases in association with von Hippel–Lindau (VHL) disease, a genetic disorder characterized by tumor and cyst development in the CNS, retina, kidneys, pancreas, and adrenal glands.^2–4^ sHBs represent 13%–50% of CNS hemangioblastomas and are found in 60%–80% of VHL patients.^3,5–9^ While sporadic cases are less common, they are clinically important due to their potential for neurological impairment. The impact of VHL disease on local tumor control remains uncertain, with conflicting findings in the literature.^4,10^

The pathophysiology of sHBs involves the proliferation of vascular endothelial and stromal cells, forming well-circumscribed nodules and cysts within the spinal cord, leading to mass effects and symptoms like pain, sensory deficits, and motor weakness, significantly affecting the patient’s quality of life.^11^ Untreated, these tumors lead to progressive neurological decline, underscoring the need for timely diagnosis and intervention.^12^

Surgical excision is the primary treatment, aiming for complete resection (CR) while preserving neurological function.^10^ Advances in microsurgical techniques and intraoperative neuromonitoring have improved outcomes by reducing morbidity and recurrence rates.^13^ Despite these advancements, up to 40% of patients experience immediate postoperative neurological deterioration.^14^ The role of extent of resection (EoR) in long-term tumor control and functional outcomes, particularly in solitary VHL-associated sHBs, remains underexplored in large cohorts.

This multicenter, international retrospective study provides original data on the clinical presentation, diagnostics, treatments, and neurological outcomes of sHBs. By collecting data from multiple centers, it aims to improve understanding, standardize treatment protocols, and enhance patient care for this rare yet impactful condition.

## Methods

Clinical data were collected after institutional review board approvals and sent to Leipzig University for centralized analysis. The study, approved by the Leipzig University ethics committee (No.: 382/23-ek), adhered to STROBE guidelines for observational cohort studies. Supplementary Methods 1 provides details on the study protocol, population, imaging definitions, endpoints, and statistical analysis.

### Study Population

The study group investigators searched institutional databases at 13 neuro-oncological centers across Europe and the United States for patients surgically treated for sHBs from January 1997 to August 2023 (Supplementary Figure 1). Inclusion criteria for patient selection were: (1) histopathological confirmation of intradural sHB; (2) age ≥18 years at diagnosis. Patients were consecutively treated at the participating centers without additional selection criteria to minimize potential biases. Standardized demographic, clinical, pathological, and imaging data were extracted from the databases for analysis (Supplementary Table 1).

### Imaging Definitions

For our study, we define the cervicothoracic junction as spanning from C7 to T4. For the thoracolumbar (T10–L2) and lumbosacral (L5–S1) transitions, we adhere to standard anatomical definitions. The distinction between a cyst and a syrinx is based on the criteria established by Chu et al.^15^ According to this definition, a syrinx is a cystic cavity extending beyond a single vertebral segment and containing fluid. In contrast, a peritumoral cyst is defined as a cystic cavity adjacent to the tumor, confined within 1 vertebral segment, and lacking enhancement. MR images were further evaluated whether preoperative bleeding occurred, intra- or extramedullary location, number of involved segments, and number of spinal lesions.

### Clinical Data Collection

Clinical data, including age, sex, sporadic or VHL-associated tumors, and clinical symptoms were anonymously extracted from patient records. Functional status was evaluated using the Karnofsky performance status (KPS) and the modified McCormick Scale (mMCS), which grades mobility as follows: grade I, normal gait; grade II, mild gait disturbance not requiring support; grade III, gait with support; grade IV, assistance required; and grade V, wheelchair dependence.^16^ These scales were assessed at several time points (preoperative, discharge, 3 months and 12 months after surgery) to investigate functional outcome. The surgical approach (laminectomy, hemilaminectomy, laminoplasty, decompression with dorsal instrumentation), EoR, and adjuvant therapy (eg, radiation therapy, chemotherapy, immunotherapy with vascular endothelial growth factor [VEGF] antibody) were documented from the patient records. Local tumor progression was defined as either the recurrence of the tumor at the same site after CR or regrowth following partial resection. Contrast-enhanced MR imaging was reviewed by the radiologists and neurosurgeons at the respective local centers. CR was defined as the complete resection of the tumor without residual tumor on MRI. Postoperative data, including the administration of adjuvant radiotherapy, presence of recurrence, and the time to recurrence or final follow-up after surgery, were also collected.^10^ To assess functional outcomes following surgery, the change in the mMCS from the preoperative period to 1 year after surgery was analyzed for each patient.

### Statistics

Statistics were analyzed using Pearson’s chi-squared test for nominal variables and Student’s *t*-test for metric variables, with all *P*-values reported as 2-sided. Univariate Cox regression and log-rank tests assessed local tumor progression, including variables with *P*-values < .10 in the multivariate Cox regression for progression-free survival (PFS).^17^ Intramedullary location and preoperative bleeding were also included because of their confounding effect regarding achievement of complete resection. Effect measures include hazard ratios (HRs) with 95% confidence intervals (CIs). Age was dichotomized according to the median value for the predefined endpoints.^18^ Kaplan–Meier PFS charts were created using Survminer and Survival packages in R version 4.3.1 (R Foundation for Statistical Computing). Bubble plot charts were created using the R package ggplot2. Staked bar charts and forest plots were created using Python’s Matplotlib package. Functional outcomes of surgically treated patients with solitary sHBs were visualized via Sankey plots. Follow-up at 12 months defined outcomes: the G-group (McCormick Scale grades I–III or stable grades) and the P-group (≥1-grade decline or remaining in grades IV/V).^19^ A directed acyclic graph (see Supplementary Figure 2) informed the multivariable binary logistic regression model of functional outcomes, adjusting for key confounders while avoiding bias. Results were visualized with forest plots.^20^

## Results

### Patient Characteristics

**Figure 1.**
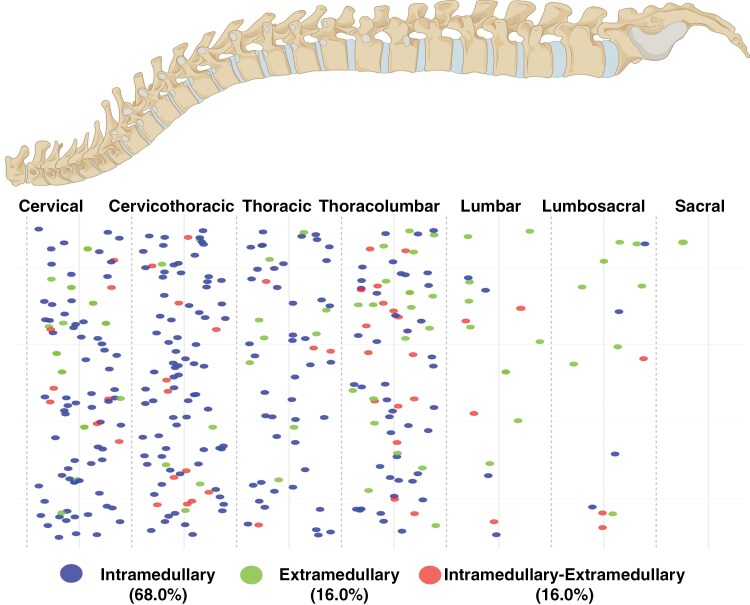
Schematic representation of the spinal column shows the distribution of spinal hemangioblastomas across different spinal levels. The dots represent individual tumors, categorized by their anatomical location: intramedullary (68.0%), extramedullary (16.0%), and intramedullary–extramedullary (16.0%).

The study cohort included 357 patients, with 89.4% having primary tumors and 10.6% recurrent tumors. The median age was 43 years (interquartile range [IQR]: 30.0–55.0), with 52.4% male. In total, 158 patients (44.3%) had sporadic sHB and 199 patients (55.7%) had VHL-associated sHB (see Supplementary Figure 3). Tumor location was mainly intramedullary (68.0%), with extramedullary (16.0%) and combined involvement (16.0%). The cervical spine was the most common site (47.3%), followed by thoracic (28.3%), cervicothoracic (8.4%), thoracolumbar (8.1%), lumbar (5.6%), and lumbosacral (2.2%) (Figure 1). Cysts were found in 42.9% of patients, and syrinx formation in 47.3%. Preoperative bleeding occurred in 5.3% of cases. Surgical approaches included laminectomy (41.2%), hemilaminectomy (46.5%), laminoplasty (10.9%), and laminectomy with dorsal instrumentation (1.4%). CR was achieved in 87.7% of cases (see Supplementary Figure 3). Adjuvant therapy was given to 9.5% of patients, with 6.2% receiving radiotherapy, 2.5% chemotherapy, and 0.8% VEGF treatment. Further details are summarized in Table 1.

**Table 1.** Patient Characteristics

Variable	All Patients (*n* = 357)
Tumor status	
Primary	319 (89.4%)
Recurrence	38 (10.6%)
Sex	
Female	170 (47.6%)
Male	187 (52.4%)
Age	
Median age	43 (IQR: 30.0–55.0)
Location^a^	
Intramedullary	242 (68.0%)
Extramedullary	57 (16.0%)
Combined	57 (16.0%)
Number of involved segments	
1	189 (52.9%)
2	104 (29.1%)
3	47 (13.2%)
4	17 (4.8%)
Spinal level	
Cervical	169 (47.3%)
Cervicothoracic	30 (8.4%)
Thoracic	101 (28.3%)
Thoracolumbar	29 (8.1%)
Lumbar	20 (5.6%)
Lumbosacral	8 (2.2%)
Cyst	
Yes	153 (42.9%)
No	204 (57.1%)
Syrinx	
Yes	169 (47.3%)
No	188 (52.7%)
Preoperative bleeding	
Yes	19 (5.3%)
No	338 (94.7%)
Extent of resection	
Complete resection	313 (87.7%)
Incomplete resection	44 (12.3%)
Surgical approach	
Laminectomy	147 (41.2%)
Laminoplasty	39 (10.9%)
Hemilaminectomy	166 (46.5%)
Laminectomy and dorsal instrumentation	5 (1.4%)
Adjuvant therapy	
No adjuvant therapy	323 (90.5%)
Radiotherapy	22 (6.2%)
Chemotherapy	9 (2.5%)
VEGF treatment	3 (0.8%)

Abbreviations: IQR, interquartile range; VEGF, vascular endothelial growth factor.

^a^Unknown in 1 case.

### Local Tumor Control After Surgery for sHB

Follow-up data for local PFS was available in 313 patients. The median follow-up time was 43.5 months (IQR: 13.0–108.3 months), while the mean follow-up time was 67.4 months (SD: 66.3 months), reflecting a right-skewed distribution due to a small number of patients with extended follow-up durations. The univariable Cox regression analysis for PFS included several key factors (see Table 2). Age (<43 vs. ≥43) was associated with an HR of 1.93 (95% CI: 0.98–3.80, *P* = .06). The KPS (≤70 vs. >70) showed a similar HR of 1.94 (95% CI: 0.98–3.84, *P* = .06). Sex (male vs. female) was not significantly associated with local PFS, with an HR of 1.07 (95% CI: 0.55–2.08, *P* = .84). Genetic disorder (von Hippel–Lindau vs. sporadic) demonstrated a trend toward significance with an HR of 2.10 (95% CI: 0.86–5.08, *P* = .10). Tumor status (recurrent vs. primary) had an HR of 2.11 (95% CI: 0.88–5.09, *P* = .10). The number of lesions (multiple vs. solitary) was significantly associated with PFS, with an HR of 2.76 (95% CI: 1.39–5.48, *P* = .004). The presence of a cyst (present vs. absent) was not significantly associated with PFS, with an HR of 1.25 (95% CI: 0.63–2.48, *P* = .52). EoR (incomplete resection vs. complete resection) was a significant factor, with an HR of 5.64 (95% CI: 2.82–11.28, *P* < .001). Variables with *P*-values < .10 were included in the multivariable Cox regression analysis (see Table 2).

**Table 2.** Univariable Cox Regression Analysis of Progression-Free Survival

Variable	Univariable		
	HR	95% CI	*P*-Value
Age	1.93	0.98–3.80	.06
(<43 vs. ≥43)			
KPS (≤70/>70)	1.94	0.98–3.84	.06
Sex (male vs. female)	1.07	0.55–2.08	.84
Genetic disorder (von Hippel–Lindau vs. sporadic)	2.10	0.86–5.08	.10
Tumor status (recurrent vs. primary)	2.11	0.88–5.09	.10
No. of lesions (multiple vs. solitary)	2.76	1.39–5.48	.004
Cyst (present vs. absent)	1.25	0.63–2.48	.52
Extent of resection (incomplete resection vs. complete resection)	5.64	2.82–11.28	.001

Abbreviations: CI, confidence interval; HR, hazard ratio; KPS, Karnofsky performance status.

**Figure 2.**
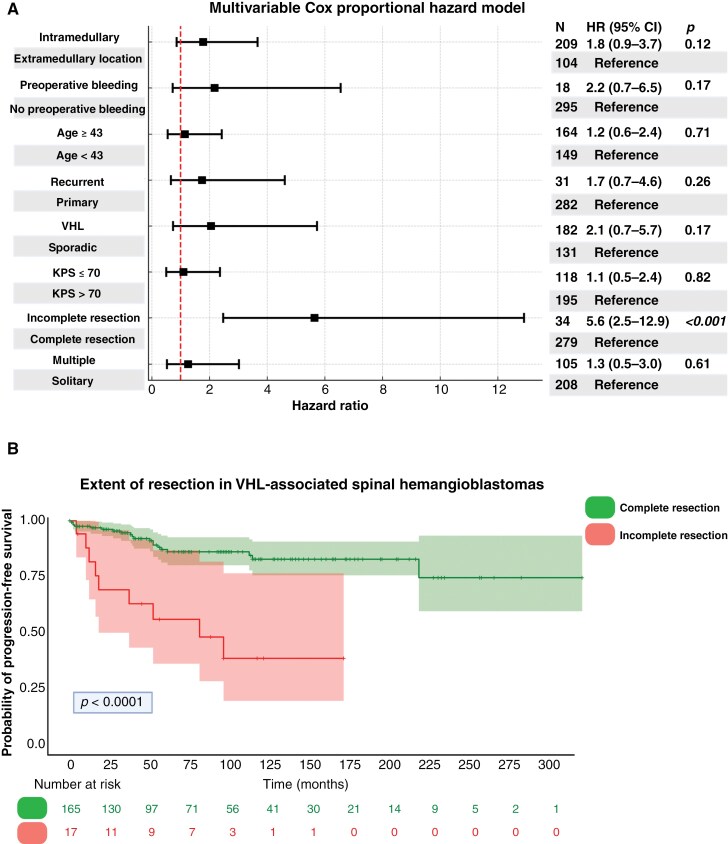
Multivariable Cox regression analysis and Kaplan–Meier survival curves for spinal hemangioblastomas. (A) Forest plot summarizing the multivariable Cox proportional hazards model for local progression-free survival (PFS). Incomplete resection was the only significant predictor of worse PFS with a hazard ratio (HR) of 6.2 (95% CI: 2.7–14.0, *P *< .001). (B) Kaplan–Meier curve demonstrating the impact of EoR on PFS in VHL-associated spinal hemangioblastomas. Patients undergoing CR had significantly better PFS compared to those with non-CR (*P *< .0001). Abbreviations: CR, complete resection; EoR, extent of resection; VHL, von Hippel–Lindau.

The multivariable Cox regression analysis of PFS identified EoR as the only independent predictor of local PFS (HR: 5.6, 95% CI: 2.5–12.9, *P* < .001) (see Figure 2A). Overall, the mean local PFS time across all patients was 260.6 months (95% CI: 239.4–281.8). The mean local PFS time for patients with sporadic sHBs was at 254.7 months (95% CI: 235.5–273.9) and for those with VHL-associated sHBs at 251.5 months (95% CI: 227.0–276.1) (see Supplementary Figure 4). The 12-month cumulative local PFS proportion was 97.1% for sporadic cases and 96.0% for VHL-associated cases. At 36 months, PFS remained high at 95.7% for sporadic cases compared to 91.4% in the VHL group. By 72 months, the local PFS proportion decreased to 90.3% for sporadic cases and 80.6% for VHL-associated cases. These findings indicate that while local PFS proportion is initially comparable, long-term outcomes at 6 years after surgery tended to be worse in the VHL group.

The impact of EoR was further investigated in VHL-associated sHBs. The local PFS analysis for VHL-associated sHBs showed that at 12 months, the PFS was 97.5% for CR and 81.6% for non-CR. By 36 months, the PFS proportion remained high at 94.6% for CR, while it dropped to 62.7% for non-CR. At 72 months, the PFS was 91.1% for CR and 55.8% for non-CR, indicating CR superior regarding long-term local tumor control in VHL-associated cases, with significantly worse outcomes observed in those with non-CR (see Figure 2B). The local PFS in sporadic sHB stratified by EoR at 12 months was 98.8% for CR and 92.3% for non-CR. By 36 months, PFS remained high at 98.7% for CR compared to 86.9% for non-CR. At 72 months, the probability of local PFS was 95.1% for CR and 65.8% for non-CR, indicating that CR provides significantly better long-term tumor control of sporadic sHB compared to non-CR, with a notably higher risk of local progression in patients who underwent non-CR (see Supplementary Figure 5).

### Functional Outcome After Surgery for sHB

**Figure 3.**
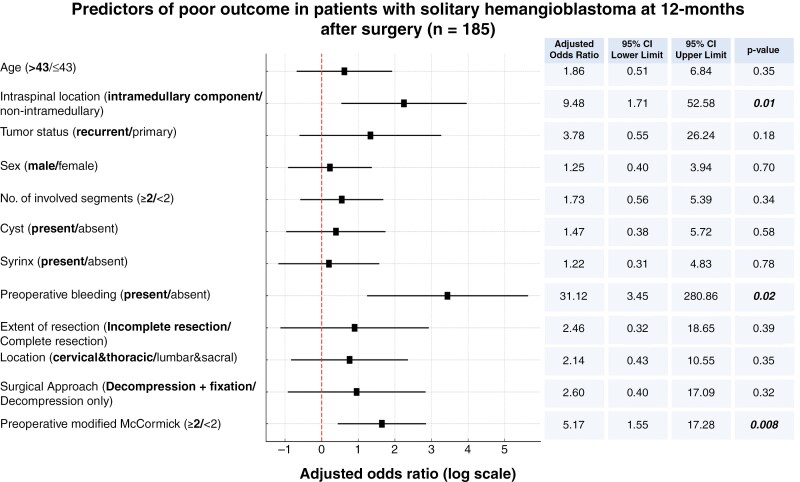
Forest plot showing predictors of poor functional outcomes at 12 months post-surgery in patients with solitary spinal hemangioblastomas. Preoperative mMCS ≥2 (OR: 5.17, *P *= .008), preoperative bleeding (OR: 31.12, *P *= .02), and intramedullary tumor location (OR: 9.48, *P *= .01) were significant predictors, while other factors like EoR and tumor location approached significance. Abbreviations: EoR, extent of resection; mMCS, modified McCormick Scale; OR = odds ratio.

The functional outcomes of sHBs were analyzed using the mMCS. The Sankey plot visualizes the transition of functional grades from preoperative assessment to 12 months postoperatively, showing that most patients experienced stability or improvement (see Supplementary Figure 6). In sporadic cases, there was a significant reduction in patients with higher McCormick grades (3 and 4) by the 12-month follow-up. Similarly, in VHL-associated cases, functional outcomes improved, with a decline in severe disability (see Supplementary Figures 7 and 8). Investigation of predictors for functional outcome was focused on a homogeneous collective with solitary sHBs in order to investigate effect of surgical treatment. Predictors of poor functional outcomes at 12 months in solitary sHB cases were identified through multivariable analysis. Preoperative functional impairment mMCS ≥2 (odds ratio [OR]: 5.17, *P* = .008), intramedullary tumor location (OR: 9.48, *P* = .01), and preoperative spontaneous bleeding (OR: 31.12, *P* = .02) were significant predictors of worse postoperative outcomes (see Figure 3) at 1 year after surgery for solitary sHB patients. These findings emphasize the importance of preoperative neurological status, intramedullary tumor location, and preoperative bleeding in determining functional prognosis, highlighting that patients with better preoperative function, non-intramedullary tumors, and no preoperative spontaneous bleeding are more likely to experience favorable long-term outcomes at 12 months after surgery.

### Patient- and Imaging-Specific Factors Being Associated With VHL-Associated sHB

**Figure 4.**
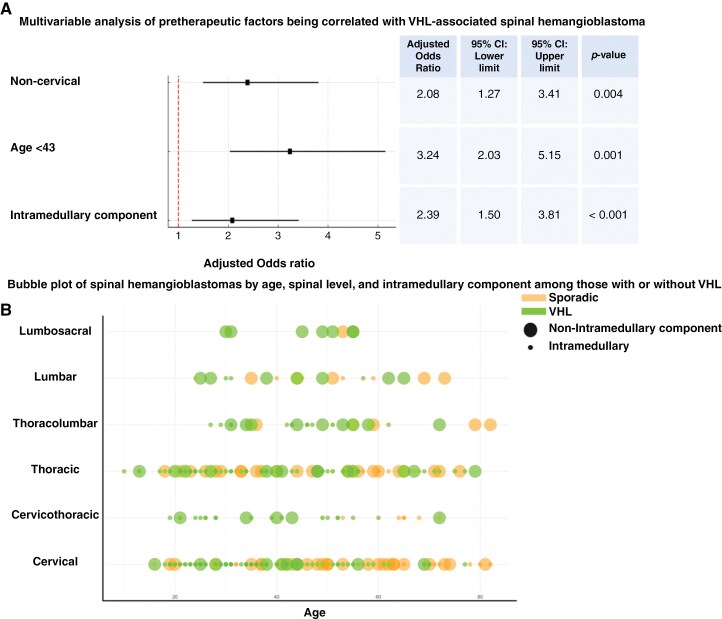
Multivariable analysis and bubble plot of factors being correlated with von Hippel–Lindau (VHL) disease in spinal hemangioblastomas. (A) Multivariable analysis of pretherapeutic factors associated with VHL-associated spinal hemangioblastoma. The forest plot shows the adjusted odds ratios (ORs) for intramedullary component, non-cervical location, and age under 43 being correlated with the presence of VHL-associated spinal hemangioblastoma. The horizontal lines represent 95% confidence intervals (CIs). The table on the right summarizes the OR, 95% CI lower and upper limits, and the *P*-value for each factor. A red dashed line indicates the OR of 1 as a reference. (B) Bubble plot of spinal hemangioblastomas by age, spinal level, and intramedullary component among those with or without VHL. The plot categorizes patients based on the presence (green) or absence (orange) of VHL. Each bubble represents a spinal hemangioblastoma, with smaller bubbles indicating those with an intramedullary component. The y-axis indicates the spinal level (from cervical to lumbosacral), while the x-axis represents individual patients’ age.

Patient- and imaging-specific factors between sporadic and VHL-associated sHBs were compared. The mean age is significantly higher in sporadic cases (49.1 ± 16.4 years) than in VHL-associated cases (39.0 ± 15.3 years, *P* = .001). Gender distribution shows a slightly higher proportion of females in VHL-associated cases (50.3% vs. 44.3%, *P* = .16). VHL-associated cases exhibit more frequent intramedullary components (73.4% vs. 61.1%, *P* = .02). Other factors like cyst presence (*P* = .36), syrinx (*P* = .34), and preoperative bleeding (*P* = .32) show no significant differences. Spinal level distribution differs, with a higher cervical involvement in sporadic cases (55.7% vs. 40.7%, *P* = .006). Supplementary Table 2 summarizes the univariable analysis. Multivariable binary logistic regression analysis of pretherapeutic factors associated with VHL-associated sHBs was performed. The significant factors of the univariable analysis were included. These factors analyzed include the presence of an intramedullary component, non-cervical tumor location, and young age at diagnosis (<43 years). The intramedullary component is significantly associated with VHL cases (OR: 2.39, 95% CI: 1.50–3.81, *P* < .001). Non-cervical location (OR: 2.08, 95% CI: 1.27–3.41, *P* = .004) and age <43 years (OR: 3.24, 95% CI: 2.03–5.15, *P* < .001) are also strongly associated with VHL cases. (see Figure 4A). Figure 4B presents a bubble plot of sHBs categorized by age, spinal level, and the presence or absence of an intramedullary component in VHL-associated and sporadic cases.

### Subgroup Analyses of the Endpoints for Primary and Recurrent sHBs

To validate the findings from the entire cohort, we performed a dedicated subgroup analysis of primary sHBs (*n* = 319) only (see Supplementary Tables 3–5, Supplementary Figures 9–14). This analysis aimed to confirm the robustness of our findings within this subset and revealed several consistent and novel insights. CR remained a pivotal predictor of prolonged local PFS in primary sHBs only. Patients undergoing complete resection demonstrated significantly better PFS compared to those with incomplete resection, with an HR of 6.26 (95% CI: 2.53–15.47, *P* < .001) from multivariable Cox regression analysis. This subgroup analysis showed preoperative bleeding as an independent factor being associated with shorter local PFS (HR: 3.47, 95% CI: 1.08–11.16, *P* = .04). Functional outcomes at 12 months after surgery were similarly consistent with the entire cohort, with better outcomes observed in patients who had intact preoperative functional status, non-intramedullary tumor location, and no preoperative bleeding. Intramedullary component, younger age, and non-cervical location were also independently correlated with VHL-associated sHBs in primary tumor patients. Further subgroup analysis for recurrent sHB only was performed in 38 patients. Local PFS analysis stratified by recurrent sporadic or VHL-associated sHBs and EoR via Kaplan–Meier method revealed no significant factors in this subgroup (see Supplementary Table 6, Supplementary Figure 15).

### Overall Survival Analysis

Overall survival (OS) was analyzed for patients with sporadic and VHL-associated sHBs. Survival data were available in 90 cases with a median follow-up of 43.5 months (IQR: 13.0–108.3). For sporadic cases, OS was 78.0% at 12 months, 68.4% at 36 months, and 56.3% at 60 months. In contrast, VHL-associated cases showed superior survival, with OS rates of 89.4% at 12 months, 82.6% at 36 months, and 75.0% at 60 months. Log-rank testing revealed no statistical significant difference (*P* = .098, see Supplementary Figure 16).

## Discussion

This international multicenter study, the largest on sHBs, highlights 3 key findings: (1) EoR is crucial for local tumor control in both sporadic and VHL-associated sHBs, as shown by multivariable Cox regression analysis. (2) Patients with preoperative neurological deficits, spontaneous tumor bleeding, or intramedullary growth have poorer neurological outcomes 1-year post-surgery. (3) Younger age at diagnosis (<43 years), intramedullary growth, and non-cervical location seem to be key indicators of VHL-associated sHBs based on preoperative patient and imaging factors.

### Local Tumor Control

Local tumor control in sHBs is strongly influenced by the EoR, with CR being critical in both sporadic and VHL-associated cases. In our study, CR was associated with significantly improved PFS in both subgroups, with PFS rates of 91.1% in VHL-associated cases and 95.1% in sporadic cases at 72 months. However, non-CR cases had much poorer outcomes, particularly in VHL-associated tumors, where PFS dropped to 55.8%. These results align with previous reports indicating that although CR offers durable control, VHL-associated tumors have higher recurrence rates due to their aggressive nature.^4^ Lifelong surveillance is, therefore, essential for managing local recurrence, particularly in younger patients with sHB.

In a study of 168 cases, the CR rate was 92.9%, with a low recurrence rate of 3.6%, which contrasts with the higher recurrence rate of 12.1% found in the present series of 313 patients.^10,21^ Prior studies also show lower CR rates in VHL-associated hemangioblastomas than in sporadic cases, contributing to higher recurrence. For example, Siller et al.^12^ reported CR rates of 100% for sporadic and 90% for VHL cases, while Yousef et al.^4^ reported 86.4% and 70%, respectively. Our CR rate of 87.7% aligns with these findings, with similar rates in both sporadic (87.3%) and VHL-associated (87.9%) cases.

### Functional Outcome After Surgery

The results from our study highlight the challenges in achieving consistent functional improvement following surgery for sHB, particularly in patients with VHL disease. Significant functional gains were observed primarily in the sporadic group, both in the short- and long-term, contrasting with the more modest improvements in VHL cases. This discrepancy could be linked to poorer preoperative functional status in VHL patients due to the cumulative burden of CNS tumors or multiple spinal tumors. Furthermore, the higher rate of new lesion development in VHL cases during follow-up (42% over 60 months) likely contributes to the limited functional recovery.^4^ Against this backdrop, we evaluate long-term outcome after surgery in a homogeneous group of patients with solitary tumors at time of surgery. Intramedullary growth, impaired preoperative functional status, and spontaneous tumor bleeding were identified as independent predictors of poor outcomes 1 year after surgery. This suggests that even VHL patients with solitary tumors can achieve functional outcomes similar to sporadic cases, emphasizing the need to optimize surgical techniques through intraoperative monitoring and advanced imaging. The importance of preoperative functional status was also highlighted in a study by Butenschoen et al.,^22^ which showed that patients with higher mMCS scores deteriorated more frequently postoperatively compared to those without deficits. Our findings align with Mehta et al.,^23^ who analyzed outcomes in 108 VHL patients with sHBs, demonstrating that resection effectively preserves neurological function, with 96% of cases stable or improved postoperatively. Their identification of ventral and intramedullary tumor location as predictors of worse outcomes is corroborated in our mixed cohort study of sporadic and VHL-associated cases. Additionally, our results emphasize evaluating all hemangioblastoma patients for VHL, not limited by age, to identify genetic predispositions. Early screening and intervention improve outcomes, supporting comprehensive management strategies for this rare but impactful condition.

These findings advocate for early treatment of sHB, preferably before severe neurological deficits appear. Preoperative bleeding in sHBs is a strong predictor of poor neurological outcomes post-surgery, consistent with reports that hemorrhagic events, although rare, often result in severe, irreversible deficits.^24^ Intramedullary hemorrhages tend to cause extensive damage, leading to poor recovery prospects. Our study underscores the significance of early surgical intervention before bleeding occurs to improve functional outcomes. However, careful decision-making is essential, especially for asymptomatic VHL patients with multiple sHBs, to avoid unnecessary surgeries and potential complications.

### Differences in Sporadic and VHL-Associated sHBs

The present study found that sHBs associated with VHL disease seem to be more often observed in younger patients (<43 years at diagnosis), with non-cervical location, and with intramedullary growth component. The younger age at diagnosis aligns with findings from previous studies.^4,10,25^ Furthermore, the higher prevalence of VHL-associated sHBs in lower spinal levels was also found in the retrospective investigation of 35 patients by Takai et al.^25^ A novel finding is the higher prevalence of intramedullary tumor component among those with a VHL-associated sHB. Increased incidence of intramedullary tumor growth among VHL-associated sHB has not been reported in the literature so far and completes this identified trias of clinical and imaging factors in sHB patients being potentially associated with VHL disease. The screening for other VHL manifestations should include an MRI of the brain to detect cranial hemangioblastomas, a neuro-ophthalmologic examination for retinal hemangioblastomas, and MR imaging to identify renal cell carcinomas and pheochromocytomas.^26,27^ Additionally, recent 2022 guidelines provide updated diagnostic criteria and recommendations for VHL screening. These guidelines advocate for a low threshold in screening younger patients presenting with hemangioblastomas. They emphasize the importance of integrating clinical, familial, and genetic information for early detection. A diagnosis can be established if a patient carries a clinically actionable VHL variant with at least 1 VHL-related manifestation or has at least 2 VHL manifestations (1 being a hemangioblastoma) even in the absence of a genetic variant. Recognized VHL manifestations include hemangioblastomas in the CNS or retina, renal cell carcinoma, pheochromocytoma, pancreatic neuroendocrine tumors, and endolymphatic sac tumors. Incorporating these recommendations into routine clinical practice facilitates timely interventions, improving long-term outcomes for affected individuals.^28^

These diagnostics and the management of these patients should be considered at an experienced VHL center.^29^ VHL-associated sHB patients might benefit in future from the recently FDA-approved HIF2α inhibitor belzutifan and potential size reduction has been recently demonstrated in a cervical sHB.^30^

### Limitations

Despite being the largest cohort of this rare disease even the current study is subject to several limitations. First, the retrospective nature of the analysis may introduce selection bias and limit causal inferences regarding the factors affecting outcomes. The study spans multiple centers, leading to variability in surgical techniques, follow-up protocols, and imaging evaluations, potentially influencing the results. Additionally, despite the large cohort, specific subgroup analyses, particularly regarding sporadic versus VHL-associated cases, may suffer from limited power. The follow-up duration, while substantial, remains heterogeneous across centers, possibly impacting the assessment of long-term outcomes like recurrence and functional status. Furthermore, local tumor recurrence was not assessed centrally and was evaluated by radiologists and neurosurgeons at the local centers, potentially introducing interrater variability. Additionally, OS analysis is limited to a subscale of the cohort and a detailed analysis of OS would necessitate more data stratified by the causes of death and for further VHL-related diseases. Finally, the use of mMCS for functional evaluation may not fully capture the nuanced spectrum of neurological recovery, especially in a rare and diverse pathology like sHB.

## Conclusions

In conclusion, our study involving 357 patients demonstrated that safe complete resection is crucial for achieving superior long-term local tumor control in both sporadic and VHL-associated sHBs. Timely surgical intervention in symptomatic sporadic patients and progressive tumors is valuable, particularly when addressing the potential impact of preoperative of neurological decline or bleeding on postoperative outcomes.

## Supplementary Material

noaf041_suppl_Supplementary_Materials

## Data Availability

Coded data can be accessed upon qualified request from the corresponding authors.
