# Supplementary material for: Local tumor control and neurological outcomes after surgery for spinal hemangioblastomas in sporadic and von Hippel–Lindau disease: A multicenter study
Source: Neuro Oncol. 2025 Feb 15;27(6):1567–78. doi: 10.1093/neuonc/noaf041 (PMC12309710; doi:10.1093/neuonc/noaf041)
Supplement: noaf041_suppl_Supplementary_Materials [file noaf041_suppl_supplementary_materials.zip › supply/noaf041_suppl_Supplementary_Table_S2.docx]

| Supplementary Table 2. Univariable comparison of patient- and imaging-specific factors among sporadic and VHL-associated spinal hemangioblastomas | | | |
| --- | --- | --- | --- |
| **Variable** | **Sporadic (*n* = 158)** | **VHL-associated (*n* = 199)** | **p-value** |
| Age (mean, SD) | 49.1 +/- 16.4 | 39.0 +/- 15.3 | *0.001* |
| **Sex**  Female  Male | 70 (44.3%)  88 (55.7%) | 100 (50.3%)  99 (49.7%) | 0.16 |
| **Intramedullary component***  Present  Absent | 96 (61.1%)  61 (38.9%) | 146 (73.4%)  53 (26.6%) | *0.02* |
| **Cyst**  Present  Absent | 65 (41.1%)  93 (58.9%) | 88 (44.2%)  111 (55.8%) | 0.36 |
| **Syrinx**  Present  Absent | 76 (48.1%)  82 (51.9%) | 93 (46.7%)  106 (53.3%) | 0.34 |
| **Preoperative bleeding**  Present  Absent | 11 (7.0%)  147 (93.0%) | 8 (4.0%)  191 (96.0%) | 0.32 |
| **Spinal level**  Cervical  Cervicothoracic  Thoracic  Thoracolumbar  Lumbar  Lumbosacral | 88 (55.7)  8 (5.1%)  44 (27.8%)  7 (4.4%)  10 (6.3%)  1 (0.6%) | 81 (40.7%)  22 (11.1%)  57 (28.6%)  22 (11.1%)  10 (5.0%)  7 (3.5%) | *0.006* |
| SD, Standard deviation, * unknown in one case | | | |
