# Supplementary material for: Local tumor control and neurological outcomes after surgery for spinal hemangioblastomas in sporadic and von Hippel–Lindau disease: A multicenter study
Source: Neuro Oncol. 2025 Feb 15;27(6):1567–78. doi: 10.1093/neuonc/noaf041 (PMC12309710; doi:10.1093/neuonc/noaf041)
Supplement: noaf041_suppl_Supplementary_Materials [file noaf041_suppl_supplementary_materials.zip › supply/noaf041_suppl_Supplementary_Table_S3.docx]

**Supplementary table 3** summarizes the main patient-, disease, and treatment-specific characteristics of patients with primary spinal hemangioblastomas.

| Supplementary table 3. Patient characteristics in primary tumors | |
| --- | --- |
| **Variable** | **Primary tumors (*n*=319)** |
| **Sex**  Female  Male | 150 (47.0%)  169 (53.0%) |
| **Age**  Mean age (SD) | 43.3 (16.5) |
| **Location***  Intramedullary  Extramedullary  Combined | 219 (68.9%)  53 (16.7%)  46 (14.5%) |
| **Number of involved segments**  1  2  3  4 | 175 (54.9%)  92 (28.8%)  40 (12.5%)  12 (3.8%) |
| **Spinal level**  Cervical  Cervicothoracic  Thoracic  Thoracolumbar  Lumbar  Lumbosacral | 157 (49.2%)  24 (7.5%)  89 (27.9%)  26 (8.2%)  17 (5.3%)  6 (1.9%) |
| **Cyst**  Yes  No | 140 (43.9%)  179 (56.1%) |
| **Syrinx**  Yes  No | 151 (47.3%)  168 (52.7%) |
| **Preoperative bleeding**  Yes  No | 18 (5.6%)  301 (94.4%) |
| **Extent of Resection**  Complete resection  Incomplete resection | 280 (87.8%)  39 (12.2%) |
| **Surgical approach**  Laminectomy  Laminoplasty  Hemilaminectomy  Laminectomy and dorsal instrumentation | 122 (38.2%)  34 (10.7%)  161 (50.5%)  2 (0.6%) |
| **Adjuvant therapy**  No adjuvant therapy  Radiotherapy  Chemotherapy  VEGF Treatment | 293 (91.8%)  14 (4.4%)  9 (2.8%)  3 (0.9%) |
| Abbreviations: * unknown in 1 case | |
|  | |
