# Supplementary material for: Local tumor control and neurological outcomes after surgery for spinal hemangioblastomas in sporadic and von Hippel–Lindau disease: A multicenter study
Source: Neuro Oncol. 2025 Feb 15;27(6):1567–78. doi: 10.1093/neuonc/noaf041 (PMC12309710; doi:10.1093/neuonc/noaf041)
Supplement: noaf041_suppl_Supplementary_Materials [file noaf041_suppl_supplementary_materials.zip › supply/noaf041_suppl_Supplementary_Table_S4.docx]

**Supplementary table 4** summarizes the univariable Cox regression analysis of local PFS in primary spinal hemangioblastomas. Extent of resection, multiple spinal hemangioblastomas, preoperative bleeding, and reduced KPS (<70) were significantly associated with local PFS in univariable Cox regression analysis.

| Supplementary table 4. Univariable Cox regression analysis of progression-free survival in primary spinal hemangioblastoma | | | |
| --- | --- | --- | --- |
| Variable | Univariable | | |
|  | HR | 95% CI | *p*-Value |
| Age | 1.85 | 0.88–3.90 | 0.11 |
| (<43 vs. **≥43**) |  |  |  |
| KPS (**≤70**/>70) | 2.34 | 1.09-5.00 | 0.03 |
| Sex  (male vs. **female**) | 1.14 | 0.55–2.38 | 0.72 |
| Location (extramedullary vs. **intramedullary**) | 1.66 | 0.77-3.59 | 0.20 |
| Preoperative bleeding (absent vs. **present**) | 4.16 | 1.58-10.98 | 0.004 |
| Genetic disorder (**Von-Hippel-Lindau** vs. sporadic) | 2.30 | 0.87-6.07 | 0.09 |
| No. of lesions (**Multiple** vs. Solitary) | 2.74 | 1.31-5.76 | 0.008 |
| Cyst (**Present** vs. Absent) | 1.21 | 0.57-2.57 | 0.61 |
| Extent of resection (**Incomplete resection** vs. Complete resection) | 6.55 | 3.09–13.88 | 0.001 |
| CI, Confidence Interval; HR, Hazard Ratio; KPS, Karnofsky Performance Status | | | |
