# Supplementary material for: Local tumor control and neurological outcomes after surgery for spinal hemangioblastomas in sporadic and von Hippel–Lindau disease: A multicenter study
Source: Neuro Oncol. 2025 Feb 15;27(6):1567–78. doi: 10.1093/neuonc/noaf041 (PMC12309710; doi:10.1093/neuonc/noaf041)
Supplement: noaf041_suppl_Supplementary_Materials [file noaf041_suppl_supplementary_materials.zip › supply/noaf041_suppl_Supplementary_Table_S5.docx]

**Supplementary table 5** summarizes the univariable comparison of patient- and imaging-specific factors among VHL-associated and sporadic primary spinal hemangioblastomas. Older age, absence of intramedullary component, and cervical location were significantly associated with sporadic tumors.

| Supplementary Table 5. Univariable comparison of patient- and imaging-specific factors among sporadic and VHL-associated primary spinal hemangioblastomas | | | |
| --- | --- | --- | --- |
| **Variable** | **Sporadic (*n* = 149)** | **VHL-associated (*n* = 170)** | **p-value** |
| Age (mean, SD) | 49.1 +/- 16.2 | 38.1 +/- 14.9 | *0.001* |
| **Sex**  Female  Male | 66 (44.3%)  83 (55.7%) | 84 (49.4%)  86 (50.6%) | 0.37 |
| **Intramedullary component***  Present  Absent | 90 (60.4%)  59 (39.6%) | 129 (76.3%)  40 (23.7%) | *0.02* |
| **Cyst**  Present  Absent | 63 (42.3%)  86 (57.7%) | 77 (45.3%)  93 (54.7%) | 0.67 |
| **Syrinx**  Present  Absent | 72 (48.3%)  77 (51.7%) | 79 (46.5%)  91 (53.5%) | 0.83 |
| **Preoperative bleeding**  Present  Absent | 11 (7.4%)  138 (92.6%) | 7 (4.1%)  163 (95.9%) | 0.31 |
| **Spinal level**  Cervical  Cervicothoracic  Thoracic  Thoracolumbar  Lumbar  Lumbosacral | 84 (56.4)  6 (4.0%)  42 (28.2%)  7 (4.7%)  9 (6.0%)  1 (0.7%) | 73 (42.9%)  18 (10.6%)  47 (27.6%)  19 (11.2%)  8 (4.7%)  5 (2.9%) | *0.016* |
| SD, Standard deviation, * unknown in one case | | | |
