# Supplementary material for: Local tumor control and neurological outcomes after surgery for spinal hemangioblastomas in sporadic and von Hippel–Lindau disease: A multicenter study
Source: Neuro Oncol. 2025 Feb 15;27(6):1567–78. doi: 10.1093/neuonc/noaf041 (PMC12309710; doi:10.1093/neuonc/noaf041)
Supplement: noaf041_suppl_Supplementary_Materials [file noaf041_suppl_supplementary_materials.zip › supply/noaf041_suppl_Supplementary_Table_S6.docx]

**Supplementary table 6**. Summary of the main patient-, disease, and treatment-specific characteristics of patients with recurrent spinal hemangioblastomas.

| Supplementary table 6 Patient characteristics in recurrent tumors | |
| --- | --- |
| Variable | **Recurrent tumors (*n*=38)** |
| Sex  Female  Male | 20 (52.6%)  18 (47.4%) |
| Age  Mean age (SD) | 45.3 (17.1) |
| Location*  Intramedullary  Extramedullary  Combined | 23 (60.5%)  4 (10.5%)  11 (29.0%) |
| Number of involved segments  1  2  3  4 | 14 (36.8%)  12 (31.6%)  7 (18.4%)  5 (13.2%) |
| Spinal level  Cervical  Cervicothoracic  Thoracic  Thoracolumbar  Lumbar  Lumbosacral | 12 (31.6%)  6 (15.8%)  12 (31.6%)  3 (7.9%)  3 (7.9%)  2 (5.3%) |
| Cyst  Yes  No | 13 (34.2%)  25 (65.8%) |
| Syrinx  Yes  No | 18 (47.4%)  20 (52.6%) |
| Preoperative bleeding  Yes  No | 1 (2.6%)  37 (97.4%) |
| Extent of Resection  Complete resection  Incomplete resection | 33 (86.8%)  5 (13.2%) |
| Surgical approach  Laminectomy  Laminoplasty  Hemilaminectomy  Laminectomy and dorsal instrumentation | 25 (65.8%)  5 (13.2%)  5 (13.2%)  3 (7.9%) |
| Adjuvant therapy  No adjuvant therapy  Radiotherapy  Chemotherapy  VEGF Treatment | 30 (78.9%)  8 (21.1%)  0 (0.0%)  0 (0.0%) |
