# Supplementary material for: Local tumor control and neurological outcomes after surgery for spinal hemangioblastomas in sporadic and von Hippel–Lindau disease: A multicenter study
Source: Neuro Oncol. 2025 Feb 15;27(6):1567–78. doi: 10.1093/neuonc/noaf041 (PMC12309710; doi:10.1093/neuonc/noaf041)

Supplementary figure 6 Sankey plot illustrating the mMCS transition from preoperative status to 12 months postsurgery in patients with spinal hemangioblastomas. The plot highlights the functional improvement or decline across different mMCS grades over time.

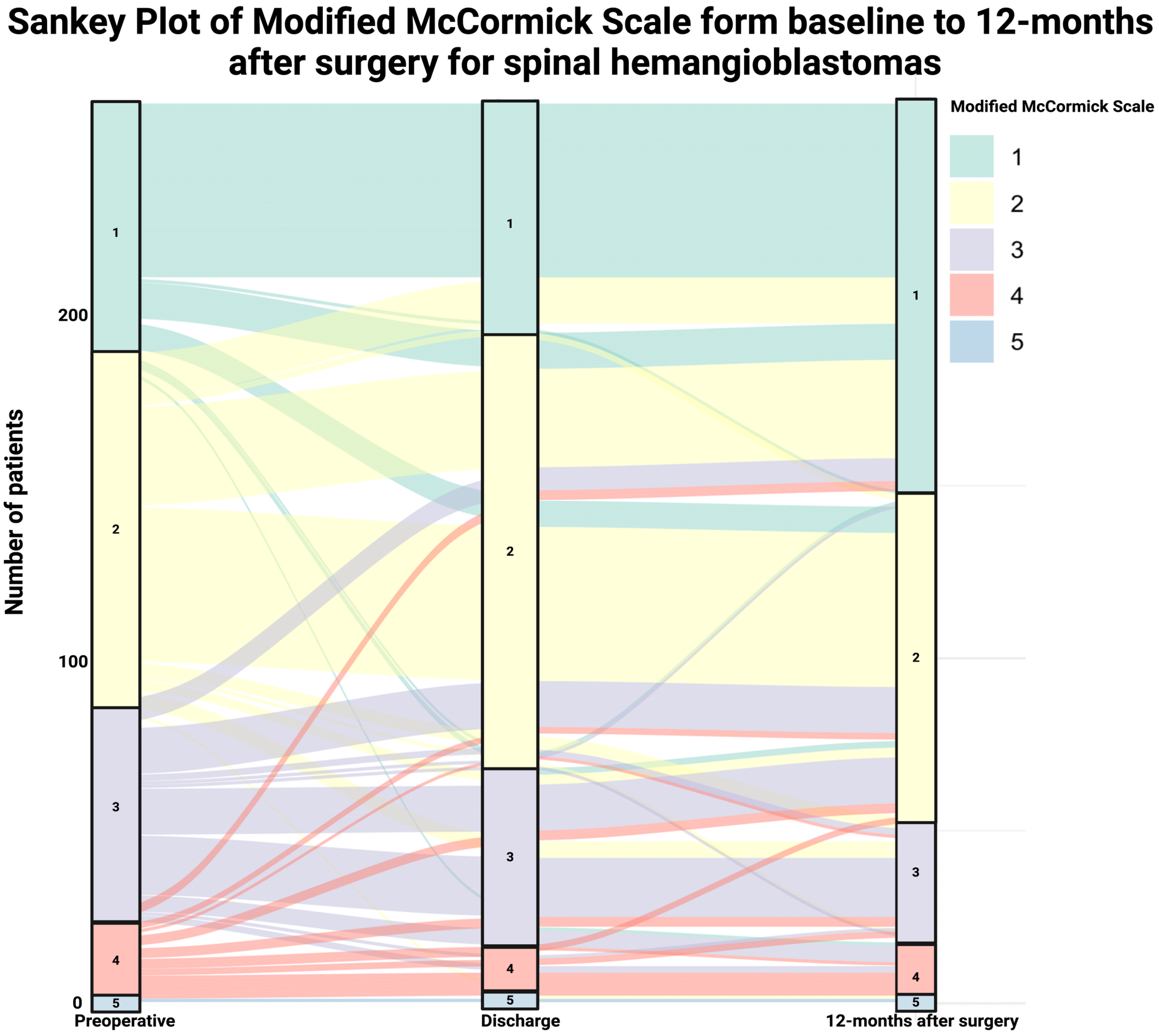

Supplement: noaf041_suppl_Supplementary_Materials [file noaf041_suppl_supplementary_materials.zip › supply/noaf041_suppl_Supplementary_Figure_S6.pdf]
