# Supplementary material for: Local tumor control and neurological outcomes after surgery for spinal hemangioblastomas in sporadic and von Hippel–Lindau disease: A multicenter study
Source: Neuro Oncol. 2025 Feb 15;27(6):1567–78. doi: 10.1093/neuonc/noaf041 (PMC12309710; doi:10.1093/neuonc/noaf041)

**Supplementary figure 7** Bar plot showing the functional status of patients with sporadic spinal hemangioblastomas before surgery, at discharge, and 12 months after surgery. Notable improvements are seen with a reduction in mMCS grades 3 and 4 postoperatively.

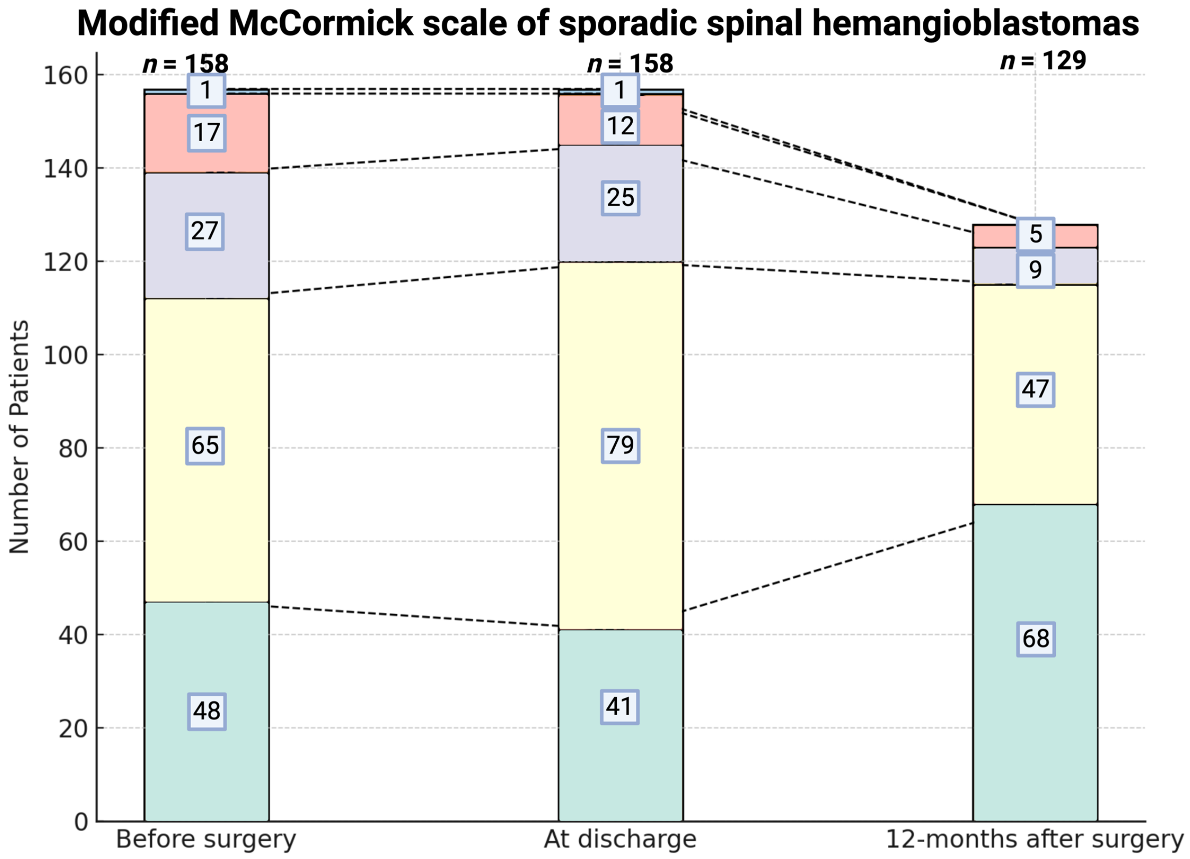

Supplement: noaf041_suppl_Supplementary_Materials [file noaf041_suppl_supplementary_materials.zip › supply/noaf041_suppl_Supplementary_Figure_S7.pdf]
