# Supplementary material for: Local tumor control and neurological outcomes after surgery for spinal hemangioblastomas in sporadic and von Hippel–Lindau disease: A multicenter study
Source: Neuro Oncol. 2025 Feb 15;27(6):1567–78. doi: 10.1093/neuonc/noaf041 (PMC12309710; doi:10.1093/neuonc/noaf041)
Supplement: noaf041_suppl_Supplementary_Materials [file noaf041_suppl_supplementary_materials.zip › supply/noaf041_suppl_Supplementary_Figure_S9.pdf]

Supplementary figure 9 shows a Kaplan-Meier curve of local PFS in primary spinal hemangioblastomas stratified by sporadic or VHL-associated primary spinal hemangioblastomas. 12-, 36-, and 60-month local PFS in primary VHL-associated spinal hemangioblastomas were 96.0%, 91.4%, and 84.9%, whereas in sporadic primary spinal hemangioblastomas the corresponding rates were 98.0%, 96.6%, and 94.2% (log-rank test: p = 0.08).

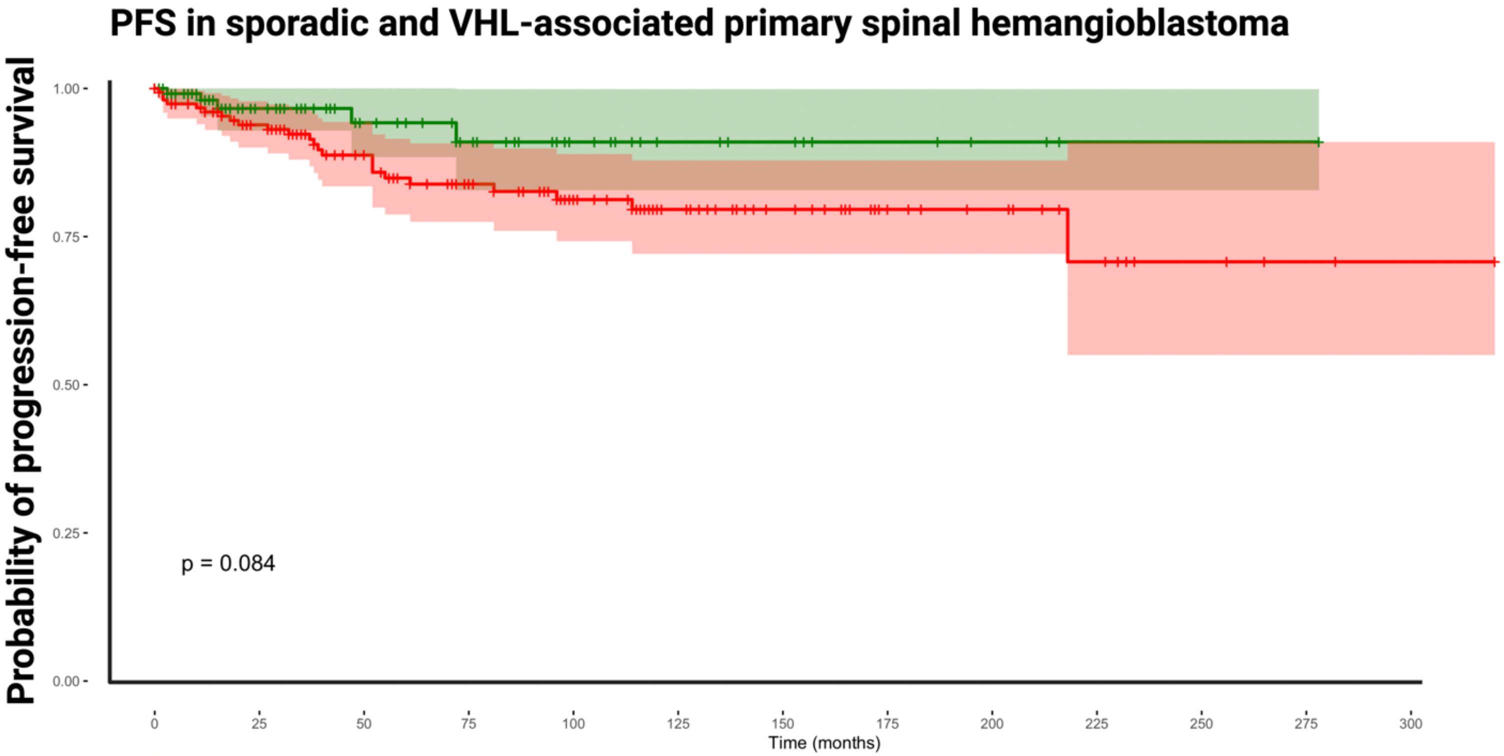

Sporadic	125	52	37	26	17	10	8	5	3	1	1	1	0
VHL	157	123	94	71	54	38	28	18	13	8	4	2	1
