# Supplementary material for: Local tumor control and neurological outcomes after surgery for spinal hemangioblastomas in sporadic and von Hippel–Lindau disease: A multicenter study
Source: Neuro Oncol. 2025 Feb 15;27(6):1567–78. doi: 10.1093/neuonc/noaf041 (PMC12309710; doi:10.1093/neuonc/noaf041)

Supplementary figure 10 shows a Kaplan-Meier curve of local PFS in primary sporadic spinal hemangioblastomas stratified by complete or incomplete resection. The 72-month local PFS in primary sporadic spinal hemangioblastomas after complete resection was 96.2%, whereas in sporadic primary spinal hemangioblastomas after incomplete resection local PFS was 65.8% (log-rank test: $p < 0.0001$).

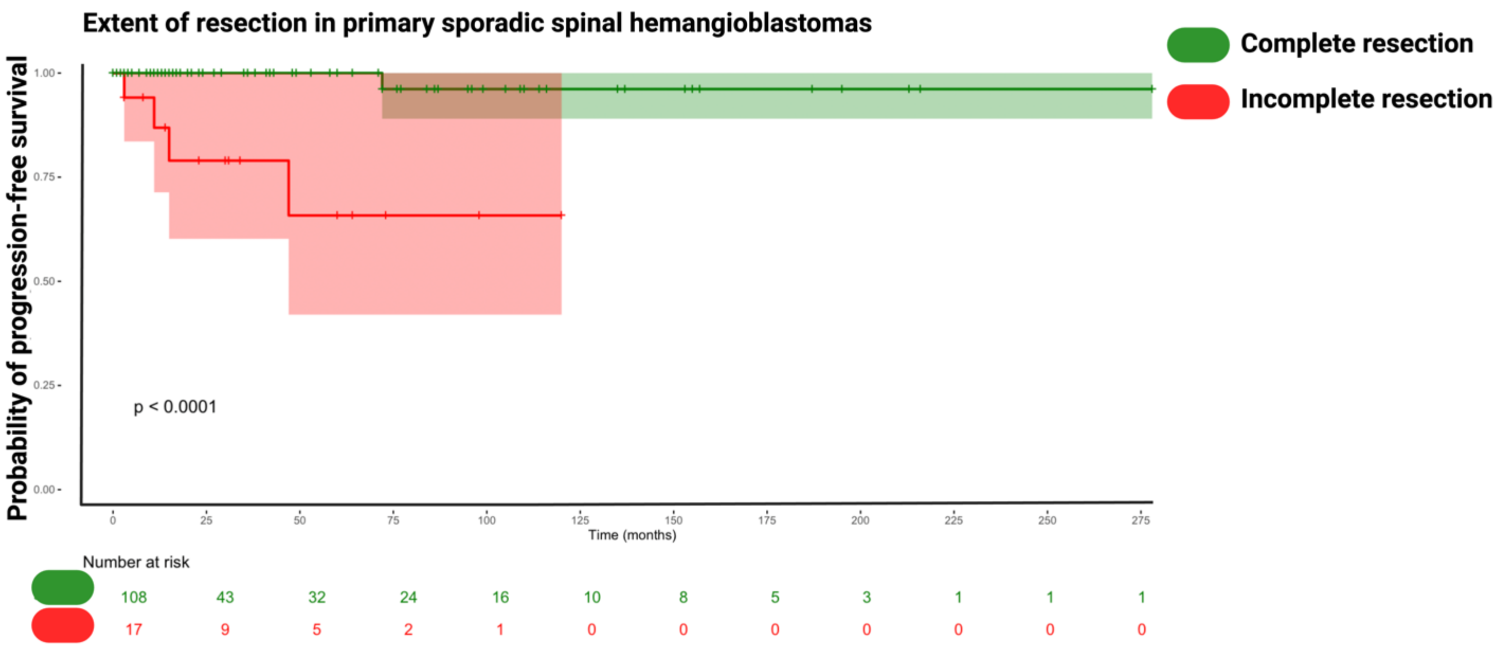

Supplement: noaf041_suppl_Supplementary_Materials [file noaf041_suppl_supplementary_materials.zip › supply/noaf041_suppl_Supplementary_Figure_S10.pdf]
