# Supplementary material for: Local tumor control and neurological outcomes after surgery for spinal hemangioblastomas in sporadic and von Hippel–Lindau disease: A multicenter study
Source: Neuro Oncol. 2025 Feb 15;27(6):1567–78. doi: 10.1093/neuonc/noaf041 (PMC12309710; doi:10.1093/neuonc/noaf041)

Supplementary figure 11 shows a Kaplan-Meier curve of local PFS in primary VHL-associated spinal hemangioblastomas stratified by complete or incomplete resection. The 12-, 36-, and 72-month local PFS rates in primary VHL-associated spinal hemangioblastomas after complete resection were 97.1%, 94.5%, and 87.1%, whereas after incomplete resection of primary VHL-associated spinal hemangioblastomas corresponding local PFS rates were 85.7%, 71.4%, and 56.3% (log-rank test: $p < 0.0001$).

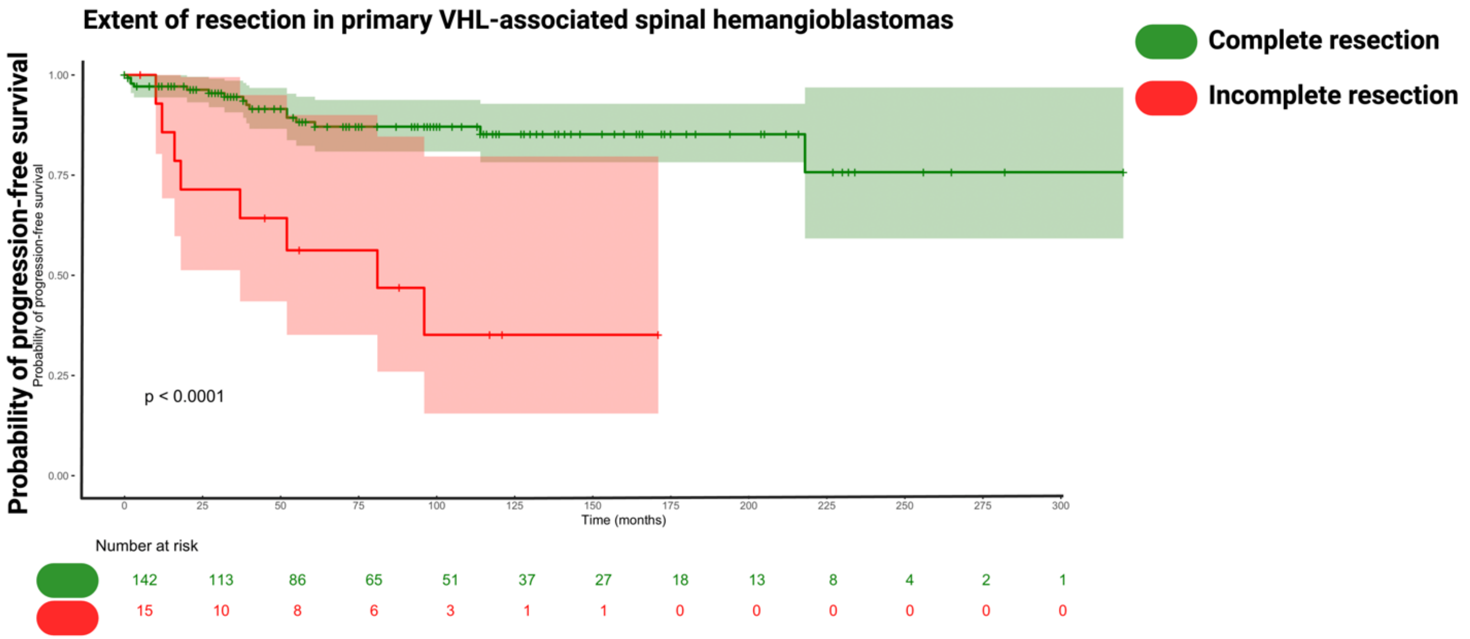

Supplement: noaf041_suppl_Supplementary_Materials [file noaf041_suppl_supplementary_materials.zip › supply/noaf041_suppl_Supplementary_Figure_S11.pdf]
