# Supplementary material for: Local tumor control and neurological outcomes after surgery for spinal hemangioblastomas in sporadic and von Hippel–Lindau disease: A multicenter study
Source: Neuro Oncol. 2025 Feb 15;27(6):1567–78. doi: 10.1093/neuonc/noaf041 (PMC12309710; doi:10.1093/neuonc/noaf041)

Supplementary figure 12 shows forest plots visualizing the results from multivariable Cox regression analysis of local PFS in primary spinal hemangioblastomas. Incomplete resection (HR: 6.26, 95% CI: 2.53-15.47, $p < 0.001$) and preoperative bleeding (HR: 3.47, 95% CI: 1.08-11.16, $p = 0.04$) were independently associated with shortened time to local tumor progression.

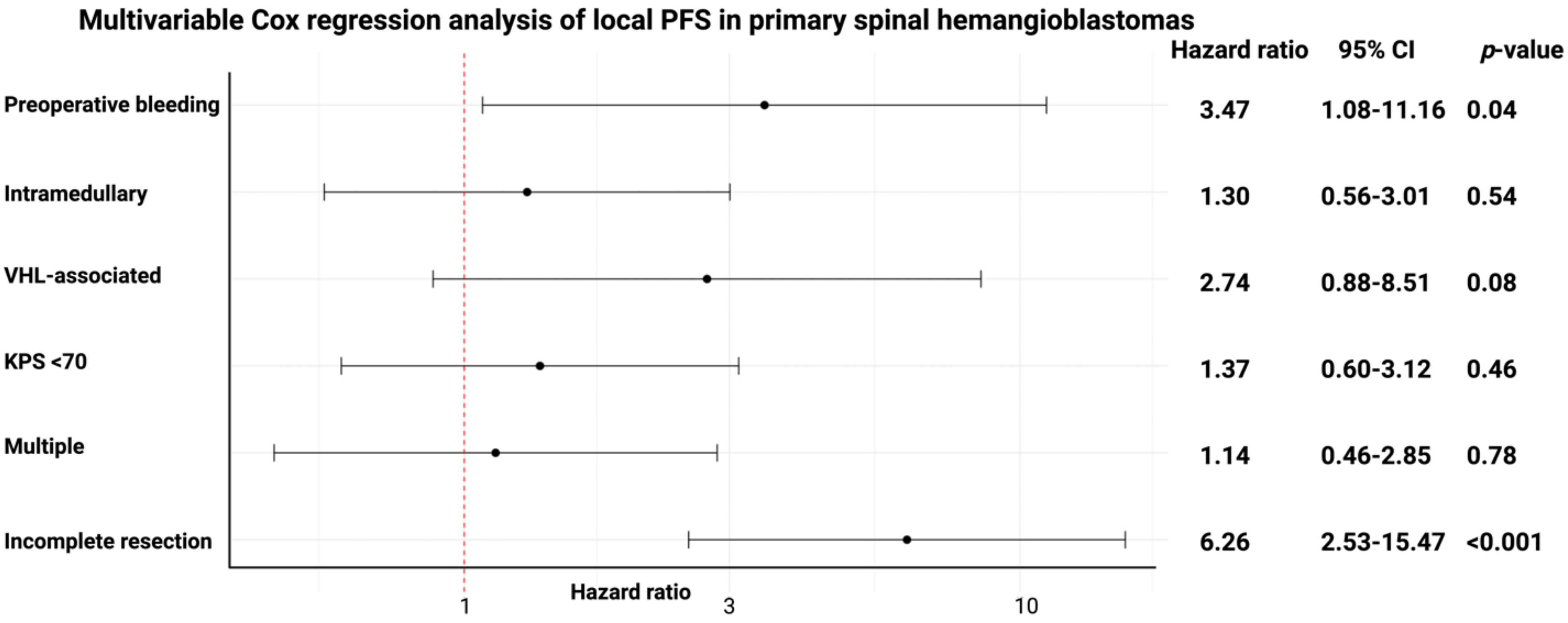

Supplement: noaf041_suppl_Supplementary_Materials [file noaf041_suppl_supplementary_materials.zip › supply/noaf041_suppl_Supplementary_Figure_S12.pdf]
