# Supplementary material for: Local tumor control and neurological outcomes after surgery for spinal hemangioblastomas in sporadic and von Hippel–Lindau disease: A multicenter study
Source: Neuro Oncol. 2025 Feb 15;27(6):1567–78. doi: 10.1093/neuonc/noaf041 (PMC12309710; doi:10.1093/neuonc/noaf041)

Supplementary figure 13 illustrates forest plots showing predictors of poor functional outcomes at 12 months post-surgery in patients with solitary primary spinal hemangioblastomas. Preoperative mMCS ≥2 (OR: 3.58, *p* = 0.046), preoperative bleeding (OR: 41.04, *p* = 0.004) and intramedullary tumor location (OR: 17.60, *p*=0.01) were significant predictors.

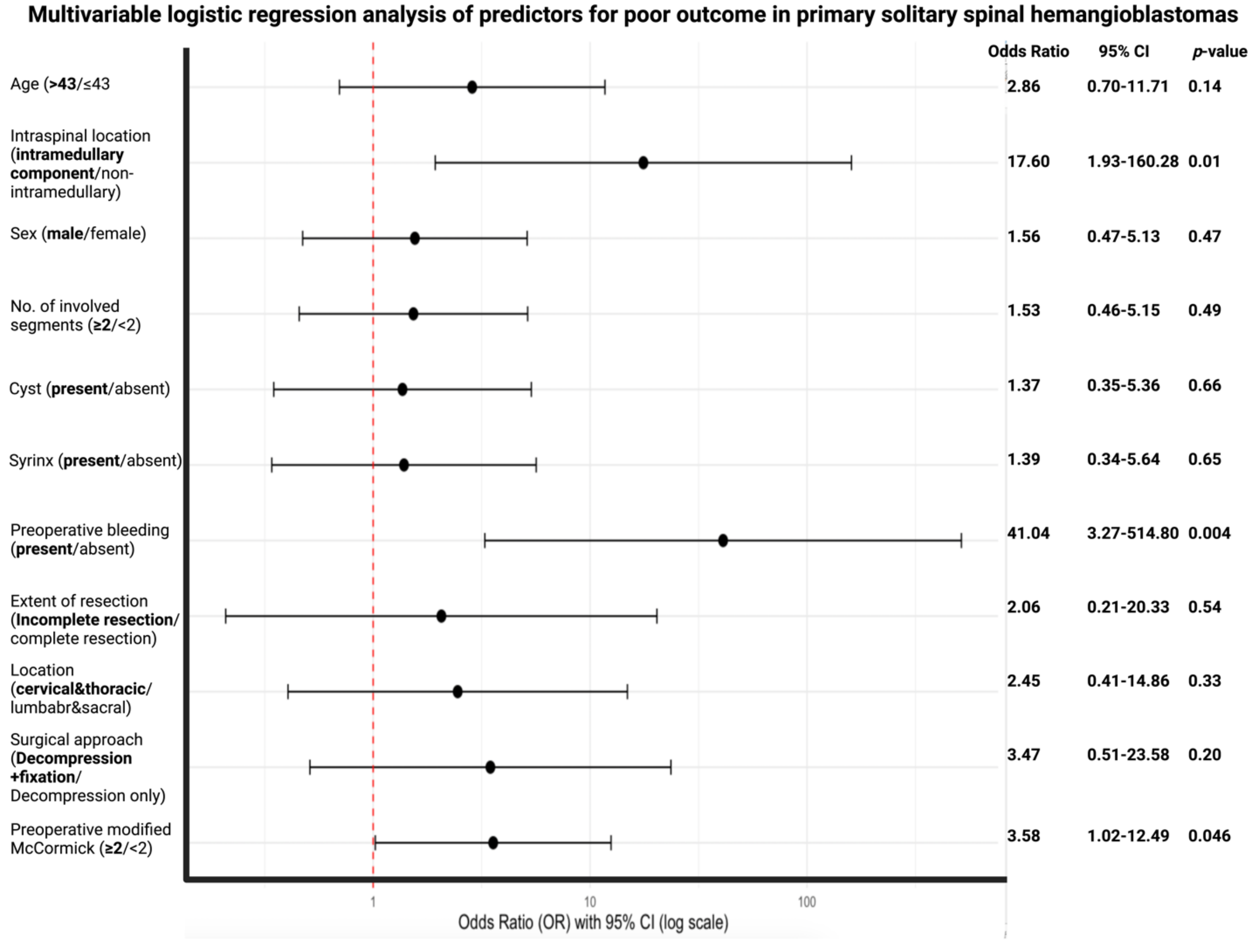

Supplement: noaf041_suppl_Supplementary_Materials [file noaf041_suppl_supplementary_materials.zip › supply/noaf041_suppl_Supplementary_Figure_S13.pdf]
