# Supplementary material for: Local tumor control and neurological outcomes after surgery for spinal hemangioblastomas in sporadic and von Hippel–Lindau disease: A multicenter study
Source: Neuro Oncol. 2025 Feb 15;27(6):1567–78. doi: 10.1093/neuonc/noaf041 (PMC12309710; doi:10.1093/neuonc/noaf041)

Supplementary figure 14 illustrates forest plots showing factors being correlated with VHL-associated primary spinal hemangioblastoma. Age<43 (OR: 3.38, $p < 0.001$), intramedullary component (OR: 2.35, $p = 0.002$), and non-cervical (OR: 2.21, $p = 0.002$) were significantly and independently correlated with VHL-associated primary spinal hemangioblastoma.

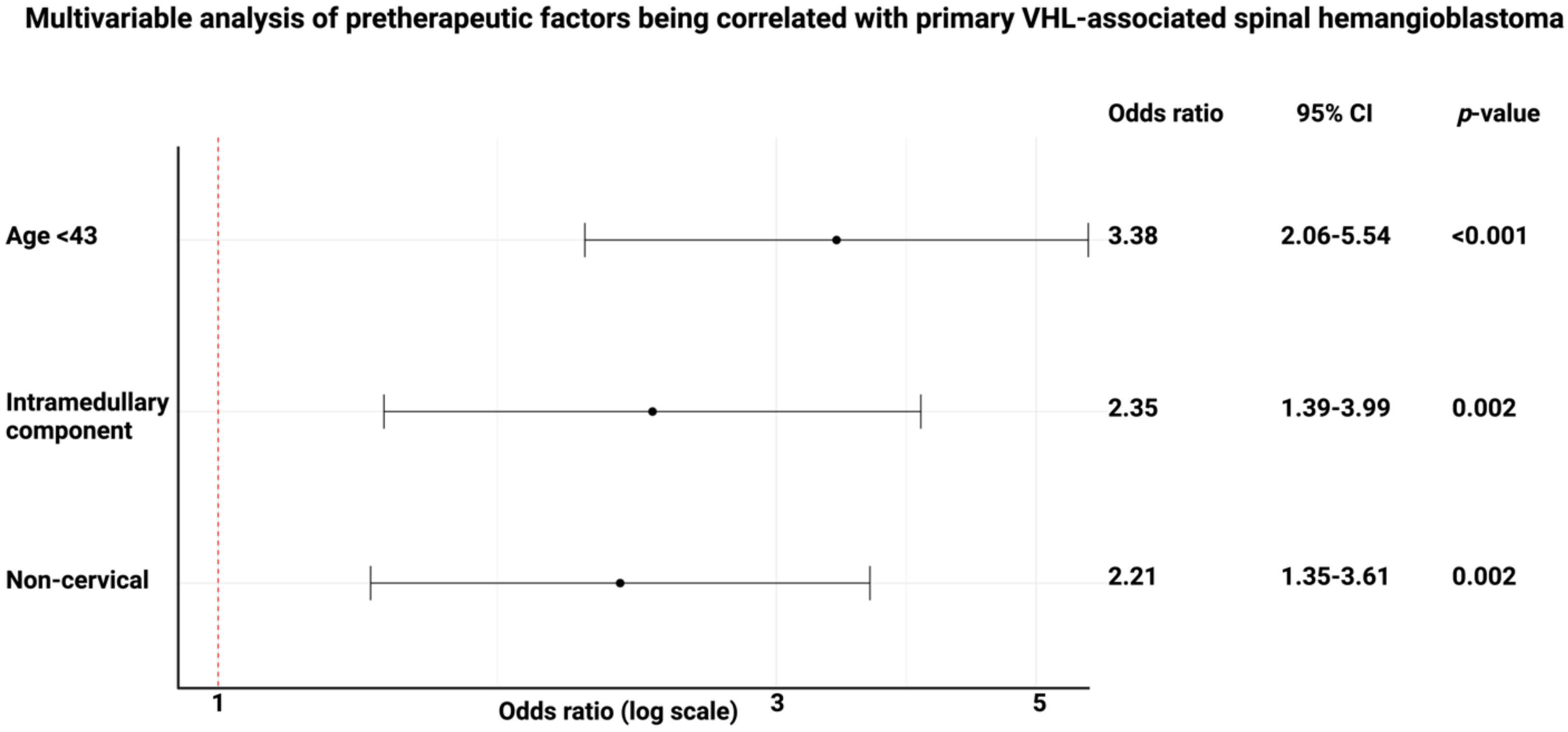

Supplement: noaf041_suppl_Supplementary_Materials [file noaf041_suppl_supplementary_materials.zip › supply/noaf041_suppl_Supplementary_Figure_S14.pdf]
