# Supplementary material for: Local tumor control and neurological outcomes after surgery for spinal hemangioblastomas in sporadic and von Hippel–Lindau disease: A multicenter study
Source: Neuro Oncol. 2025 Feb 15;27(6):1567–78. doi: 10.1093/neuonc/noaf041 (PMC12309710; doi:10.1093/neuonc/noaf041)

Supplementary figure 15 illustrates Kaplan-Meier curves of local PFS probability stratified by sporadic or VHL-associated recurrent spinal hemangioblastomas (log-rank test: $p = 0.37$) **(A)**. Furthermore, local PFS probability was also stratified by complete or incomplete resection in recurrent spinal hemangioblastomas (log-rank test: $p = 0.22$)

A

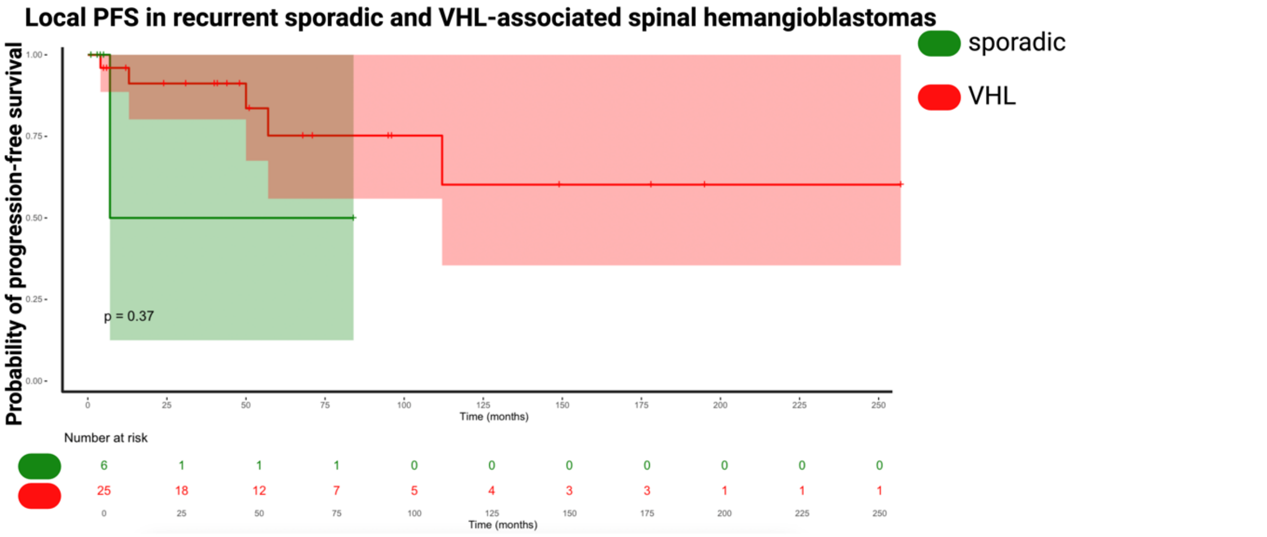

B

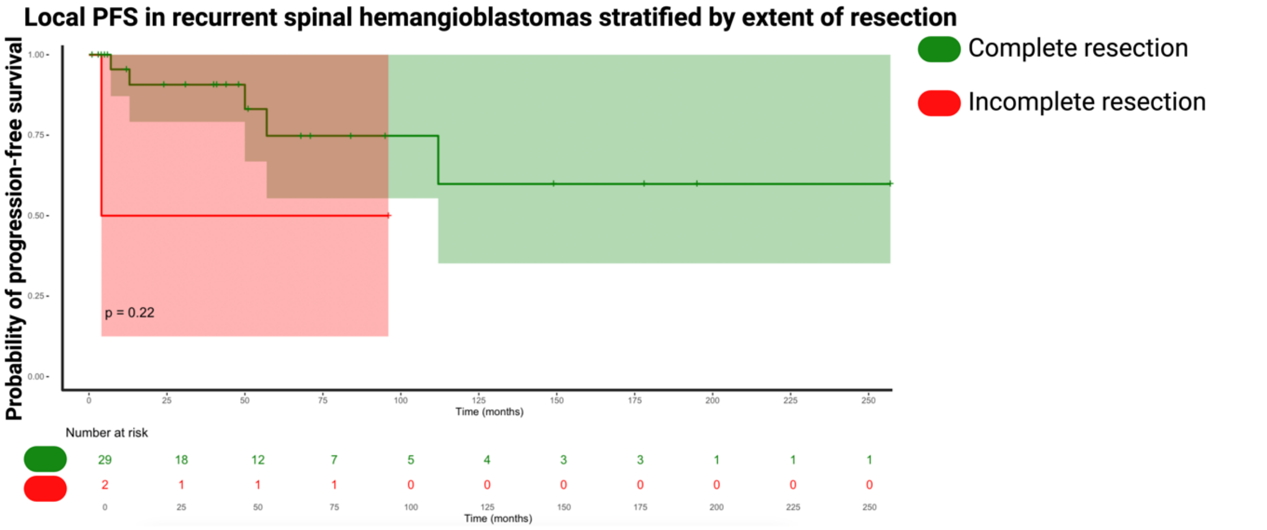

Supplement: noaf041_suppl_Supplementary_Materials [file noaf041_suppl_supplementary_materials.zip › supply/noaf041_suppl_Supplementary_Figure_S15.pdf]
