# Supplementary material for: Local tumor control and neurological outcomes after surgery for spinal hemangioblastomas in sporadic and von Hippel–Lindau disease: A multicenter study
Source: Neuro Oncol. 2025 Feb 15;27(6):1567–78. doi: 10.1093/neuonc/noaf041 (PMC12309710; doi:10.1093/neuonc/noaf041)

Supplementary figure 16 Overall survival analysis

Kaplan-Meier curve illustrating probability of survival stratified by sporadic (red) or VHL-associated (green) spinal hemangioblastoma patients. Log-rank test revealed no statistically significant difference (p = 0.098)

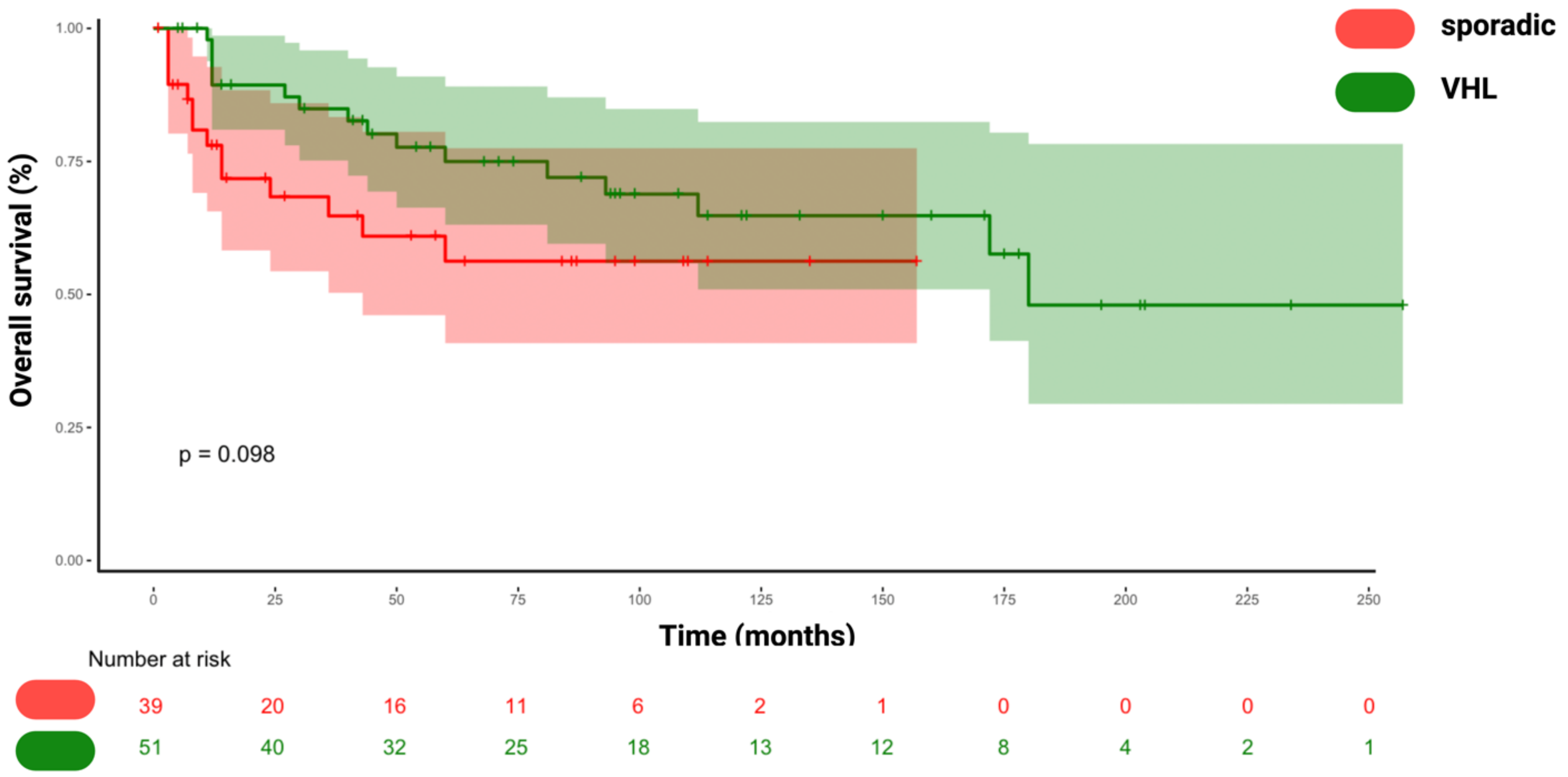

Supplement: noaf041_suppl_Supplementary_Materials [file noaf041_suppl_supplementary_materials.zip › supply/noaf041_suppl_Supplementary_Figure_S16.pdf]
