# Supplementary material for: Local tumor control and neurological outcomes after surgery for spinal hemangioblastomas in sporadic and von Hippel–Lindau disease: A multicenter study
Source: Neuro Oncol. 2025 Feb 15;27(6):1567–78. doi: 10.1093/neuonc/noaf041 (PMC12309710; doi:10.1093/neuonc/noaf041)
Supplement: noaf041_suppl_Supplementary_Materials [file noaf041_suppl_supplementary_materials.zip › supply/noaf041_suppl_Supplementary_Material.pdf]

International collaboration on spinal hemangioblastoma research

Study period:

08/01/2023 to 12/31/2025.

Aims

(1) To provide large-scale retrospective studies through the compilation of international data collected from local databases that include spinal hemangioblastoma patients, exclusively, and to use these many cohorts to cross-validate important and crucial research questions.

(2) Strengthen international collaboration on hemangioblastoma research.

Research questions

- Can patient-tailored follow-up that consider (1) extent of resection (complete resection, incomplete resection +/- radiotherapy), (2) proliferative state (Ki-67/MIB-1 index), (3) patient- (age, sex) and disease-specific characteristics (size, cystic appearance, syrinx) efficiently improve surveillance and outcome prediction of spinal hemangioblastoma patients
- What is the treatment-efficacy of gross total resection compared to subtotal resection in terms of progression-free survival for sporadic and Von-Hippel-Lindau associated hemangioblastomas?
- Is there a role of adjuvant therapy (radiation, bevacizumab) in spinal hemangioblastomas?
- What is the application of Ki-67/MIB-1 in hemangioblastomas?
- How important is it to consider competing risks to accurately establish risk of hemangioblastoma recurrence?

Background

Hemangioblastomas account for approximately 2% of all central nervous system tumors and are biologically benign vascular neoplasms. Approximately 66% of all hemangioblastomas occur sporadically, and the remaining cases grow in the context of von Hippel-Lindau disease (1). Despite the benign nature and the classification as a WHO grade 1 tumor, there are also case series reporting aggressive craniospinal dissemination of sporadic hemangioblastoma and mortality due to spinal dissemination and hemangioblastoma progression (2). The diffuse leptomeningeal dissemination is often called hemangioblastomatosis and has been described in a variety of

investigations (3-7). However, the current evidence regarding spinal hemangioblastomas is limited by a high number of single-institutional series reporting about small cohorts which do not necessarily enable to draw strong conclusions and there is the need for large-scale investigations to overcome the reported heterogeneity in spinal hemangioblastoma cohorts.
We hypothesized that compiling data from many independent cohorts would allow for attenuating limitations inherent from single cohorts.

Methods

This project comprises several meta-analyses of individual patient data (IPD) and adheres to the PRISMA-IPD Statement (8). We will apply the 'one-stage' approach, in which all received data will be compiled and analyzed simultaneously.
We include patients that have histopathologically confirmed spinal hemangioblastomas on time of entry into the respective study or database, exclusively. Surgically treated cranial hemangioblastoma patients are not eligible for this project. If the individual database contains patients with solely cranial lesions, please omit or highlight these so we can remove them.
Please indicate whether *all* are patients registered consecutively as they were admitted to your facilities, or, whether it is possible that more patients were admitted at your facilities but not registered in this database.

List of covariates:

- Primary or recurrent case
- age on diagnosis of primary hemangioblastoma
- sex
- Sporadic or von Hippel-Lindau associated tumor
- Localization
- Solitary/Multiple
- Tumor size / Imaging characteristics (Cystic appearance, Syrinx)
- Extent of resection
- Surgical treatment with or without preoperative embolization
- Neurological outcome according to modified McCormick scale
- recurrence-free survival in months (time from primary hemangioblastoma diagnosis to recurrence)
- recurrence: yes/no/lost

- Ki67/MIB-1 in the primary spinal hemangioblastoma lesion,
- If recurrence, Ki67 in that recurrent lesion (if available)
- location of progression (local, distant, leptomeningeal dissemination)
- adjuvant therapy regime (conservative, radiation, chemotherapy, VEGF antibody treatment)

Definitions

Tumor progression

Tumor progression is defined as the increase of residual tumor portion or the regrowth of new tumor portions after gross total resection. Time to progression is measured from surgical treatment to date of appointment diagnosing tumor progression.

Transition Regions in the Spine

- **Cervicothoracic Transition**: For the purposes of this study, we define the cervicothoracic transition as the region from **C7 to T4**.
- **Thoracolumbar Transition**: The thoracolumbar transition is defined from **T10 to L2**, according to widely accepted anatomical definitions.
- **Lumbosacral Transition**: The lumbosacral transition extends from **L5 to S1**, following standard anatomical guidelines.

Distinction Between Cyst and Syrinx

The differentiation between a cyst and a syrinx follows the definition provided by Chu et al. (9). According to this:

- A syrinx is a cystic cavity that is larger than a single vertebral segment and contains fluid.
- A peritumoral cyst is a cystic cavity adjacent to the tumor, confined to within one vertebral segment, and without any cyst enhancement.

External validity

We expect that this study will have high external validity and data robustness, as we expect to successfully collect validated data on >300 spinal hemangioblastoma patients from international databases in Europe, and North America.

Ethical statement

The project includes completely anonymized data, only, that in the majority of cases have been published already. This includes *no specific dates*, but only time in between events. Therefore, all patients included in this compilation will be completely unidentifiable. The project is supported by institutional biostatisticians to provide a strong statistical analysis. IRB approval was obtained by the local committee of Leipzig University (No.: 382/23-ek)

Best regards

PD Dr. med. Johannes Wach, MBA
Department of Neurosurgery
Chairman: Univ. – Prof. Dr. med. Erdem Güresir
University Hospital of Leipzig, Germany

References

1. Bamps S, Calenbergh FV, Vleeschouwer SD, Loon JV, Sciot R, Legius E, Goffin J. What the neurosurgeon should know about hemangioblastoma, both sporadic and in Von Hippel-Lindau disease: A literature review. Surg Neurol Int. 2013 Nov 8;4:145. doi: 10.4103/2152-7806.121110.
2. Dantas F, Raso JL, Braga PSG, Botelho RV, Dantas FLR. Aggressive dissemination of central nervous system hemangioblastoma without association with von Hippel-Lindau disease: A case report and literature review. Surg Neurol Int. 2022 Aug 12;13:358. doi: 10.25259/SNI_304_2022.
3. Weil RJ, Vortmeyer AO, Zhuang Z, Pack SD, Theodore N, Erickson RK, Oldfield EH. Clinical and molecular analysis of disseminated hemangioblastomatosis of the central nervous system in patients without von Hippel-Lindau disease. Report of four cases. J Neurosurg. 2002 Apr;96(4):775-87. doi: 10.3171/jns.2002.96.4.0775.
4. Reyes-Botero G, Gállego Pérez-Larraya J, Sanson M. Sporadic CNS hemangioblastomatosis, response to sunitinib and secondary polycythemia. J Neurooncol. 2012 Apr;107(2):439-40. doi: 10.1007/s11060-011-0752-9. Epub 2011 Nov 11.
5. Ramachandran R, Lee HS, Matthews B, Shatzel A, Tihan T. Intradural extramedullary leptomeningeal hemangioblastomatosis and paraneoplastic limbic encephalitis diagnosed at autopsy: an unlikely pair. Arch Pathol Lab Med. 2008 Jan;132(1):104-8. doi: 10.5858/2008-132-104-IELHAP.
6. Kato M, Ohe N, Okumura A, Shinoda J, Nomura A, Shuin T, Sakai N. Hemangioblastomatosis of the central nervous system without von Hippel-Lindau disease: a case report. J Neurooncol. 2005 May;72(3):267-70. doi: 10.1007/s11060-004-2244-7.
7. Franco A, Pytel P, Lukas RV, Chennamaneni R, Collins JM. CNS hemangioblastomatosis in a patient without von Hippel-Lindau disease. CNS Oncol. 2017 Apr;6(2):101-105. doi: 10.2217/cns-2016-0027.
8. Stewart LA, Clarke M, Rovers M, Riley RD, Simmonds M, Stewart G, Tierney JF; PRISMA-IPD Development Group (2015) Preferred Reporting Items for Systematic Review and Meta-Analyses of individual participant data: the PRISMA-IPD Statement. JAMA 313(16):1657-65. https://doi.org/10.1001/jama.2015.3656.
9. Chu BC, Terae S, Hida K, Furukawa M, Abe S, Miyasaka K. MR findings in spinal hemangioblastoma: correlation with symptoms and with angiographic and surgical findings. AJNR Am J Neuroradiol. 2001 Jan;22(1):206-17.
