# Supplementary material for: Local tumor control and neurological outcomes after surgery for spinal hemangioblastomas in sporadic and von Hippel–Lindau disease: A multicenter study
Source: Neuro Oncol. 2025 Feb 15;27(6):1567–78. doi: 10.1093/neuonc/noaf041 (PMC12309710; doi:10.1093/neuonc/noaf041)

Supplementary figure 3 *Distribution of tumor types and extent of resection.*

The upper donut chart illustrates the proportion of **VHL-associated tumors** (green, 55.7%, n = 199) and **sporadic tumors** (orange, 44.3%, n = 158) in the study cohort (n = 357).

The lower donut chart represents the extent of resection, highlighting the percentage of **complete resections** (blue, 87.7%, n = 313) versus **incomplete resections** (pink, 12.3%, n = 44) among the same cohort.

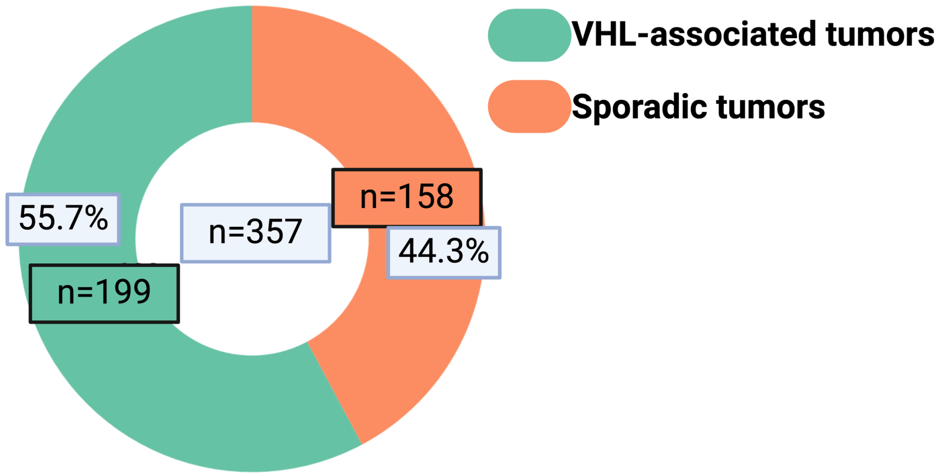

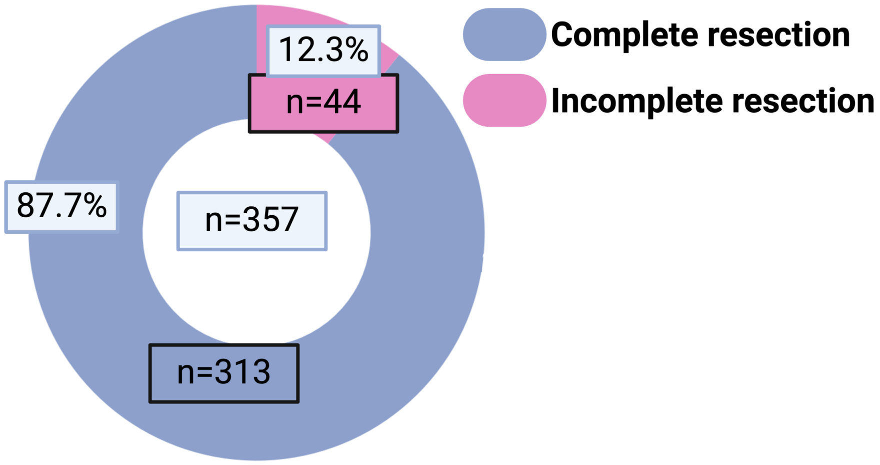

Supplement: noaf041_suppl_Supplementary_Materials [file noaf041_suppl_supplementary_materials.zip › supply/noaf041_suppl_Supplementary_Figure_S3.pdf]
