# Supplementary material for: Local tumor control and neurological outcomes after surgery for spinal hemangioblastomas in sporadic and von Hippel–Lindau disease: A multicenter study
Source: Neuro Oncol. 2025 Feb 15;27(6):1567–78. doi: 10.1093/neuonc/noaf041 (PMC12309710; doi:10.1093/neuonc/noaf041)

Supplementary figure 4 Kaplan-Meier curve comparing PFS between sporadic and VHL-associated spinal hemangioblastomas. VHL-associated cases showed a trend toward worse outcomes, though not statistically significant (p = 0.09).

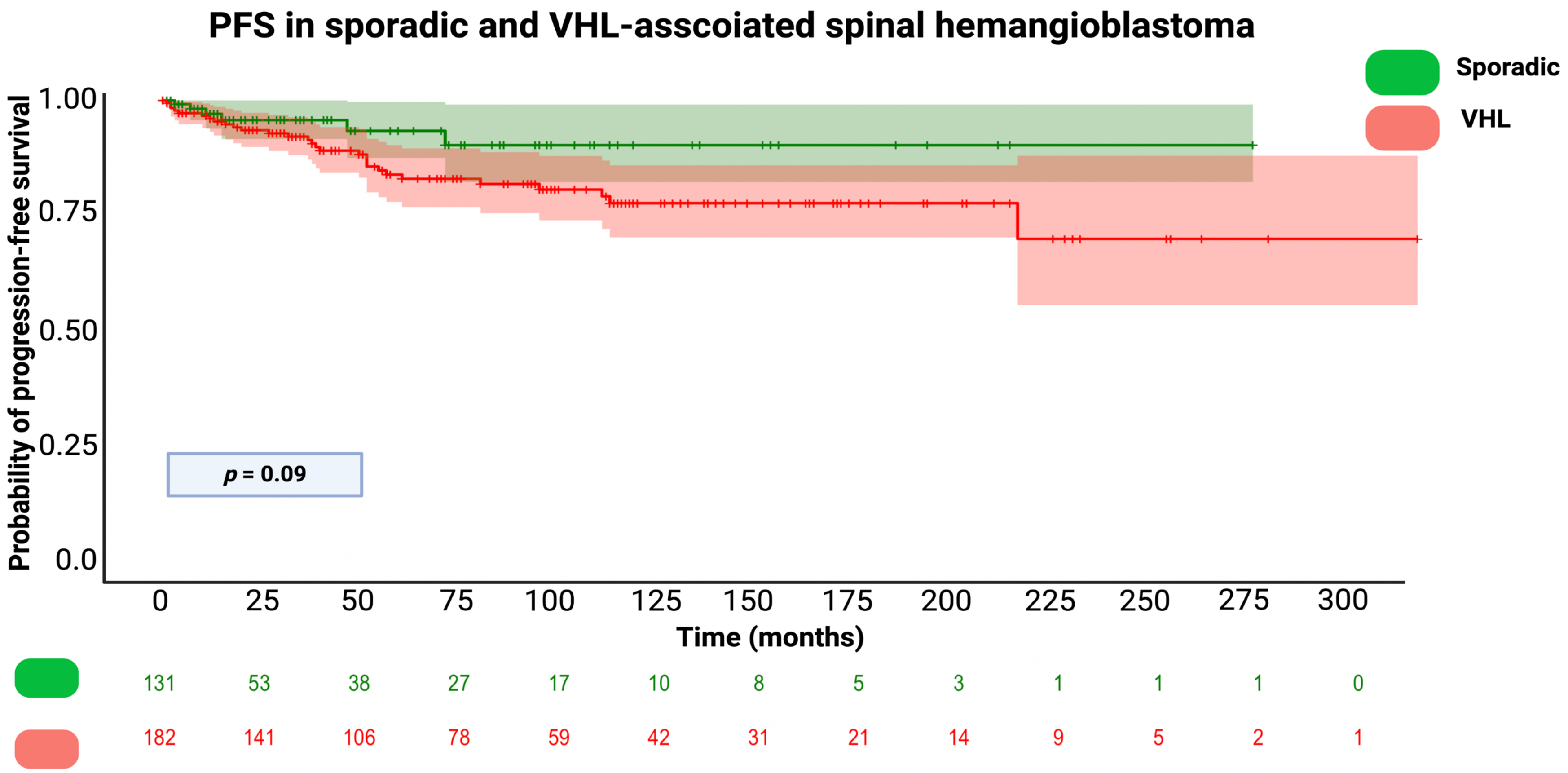

Supplement: noaf041_suppl_Supplementary_Materials [file noaf041_suppl_supplementary_materials.zip › supply/noaf041_suppl_Supplementary_Figure_S4.pdf]
