# Supplementary material for: Local tumor control and neurological outcomes after surgery for spinal hemangioblastomas in sporadic and von Hippel–Lindau disease: A multicenter study
Source: Neuro Oncol. 2025 Feb 15;27(6):1567–78. doi: 10.1093/neuonc/noaf041 (PMC12309710; doi:10.1093/neuonc/noaf041)

Supplementary figure 1 *Geographical distribution of participating neuro-oncological centers.* The map provides a schematic localization of the 13 centers contributing to the study. Red dots indicate participating institutions, with corresponding labels denoting their names. The study includes institutions from the United States (University of San Francisco, Stanford University) and Europe (Charité Berlin, University of Hamburg, University of Duisburg-Essen, Goethe University Frankfurt, Heidelberg University, University of Freiburg, Leipzig University, University of Regensburg, TU Munich, University of Innsbruck, and Medical University of Warsaw).

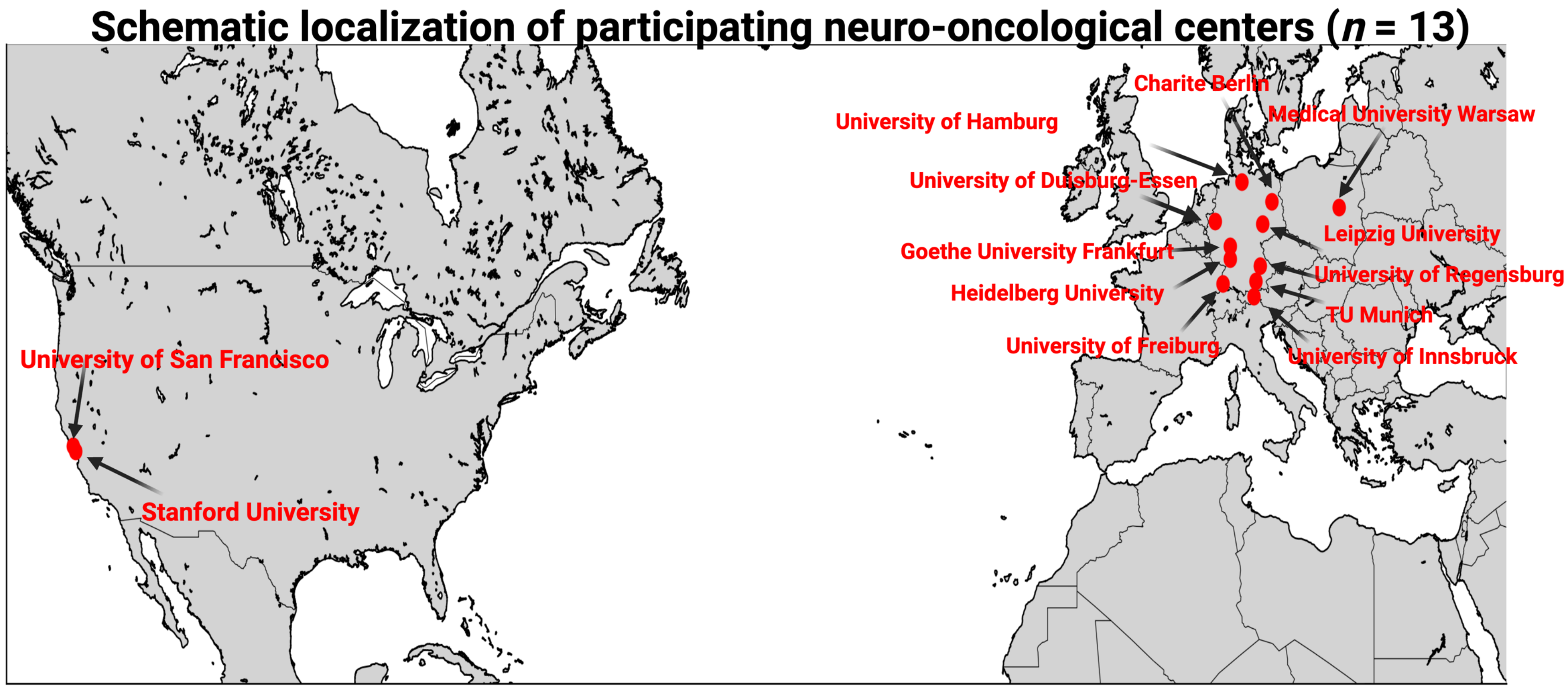

Supplement: noaf041_suppl_Supplementary_Materials [file noaf041_suppl_supplementary_materials.zip › supply/noaf041_suppl_Supplementary_Figure_S1.pdf]
