# Supplementary material for: Local tumor control and neurological outcomes after surgery for spinal hemangioblastomas in sporadic and von Hippel–Lindau disease: A multicenter study
Source: Neuro Oncol. 2025 Feb 15;27(6):1567–78. doi: 10.1093/neuonc/noaf041 (PMC12309710; doi:10.1093/neuonc/noaf041)

Supplementary figure 2 Directed Acyclic Graph (DAG) for Factors Affecting Functional Outcomes at 12 Months Post-Surgery for Solitary Spinal Hemangioblastomas.The DAG depicts key variables and their relationships impacting post-surgical outcomes. Nodes represent factors like age, extent of resection, and preoperative McCormick grade. Arrows show causal paths leading to the outcome. Important pathways include the influence of intramedullary components and resection extent. This graph guided the selection of variables for multivariable analysis to control for confounders, enhancing the study's accuracy in predicting functional recovery.

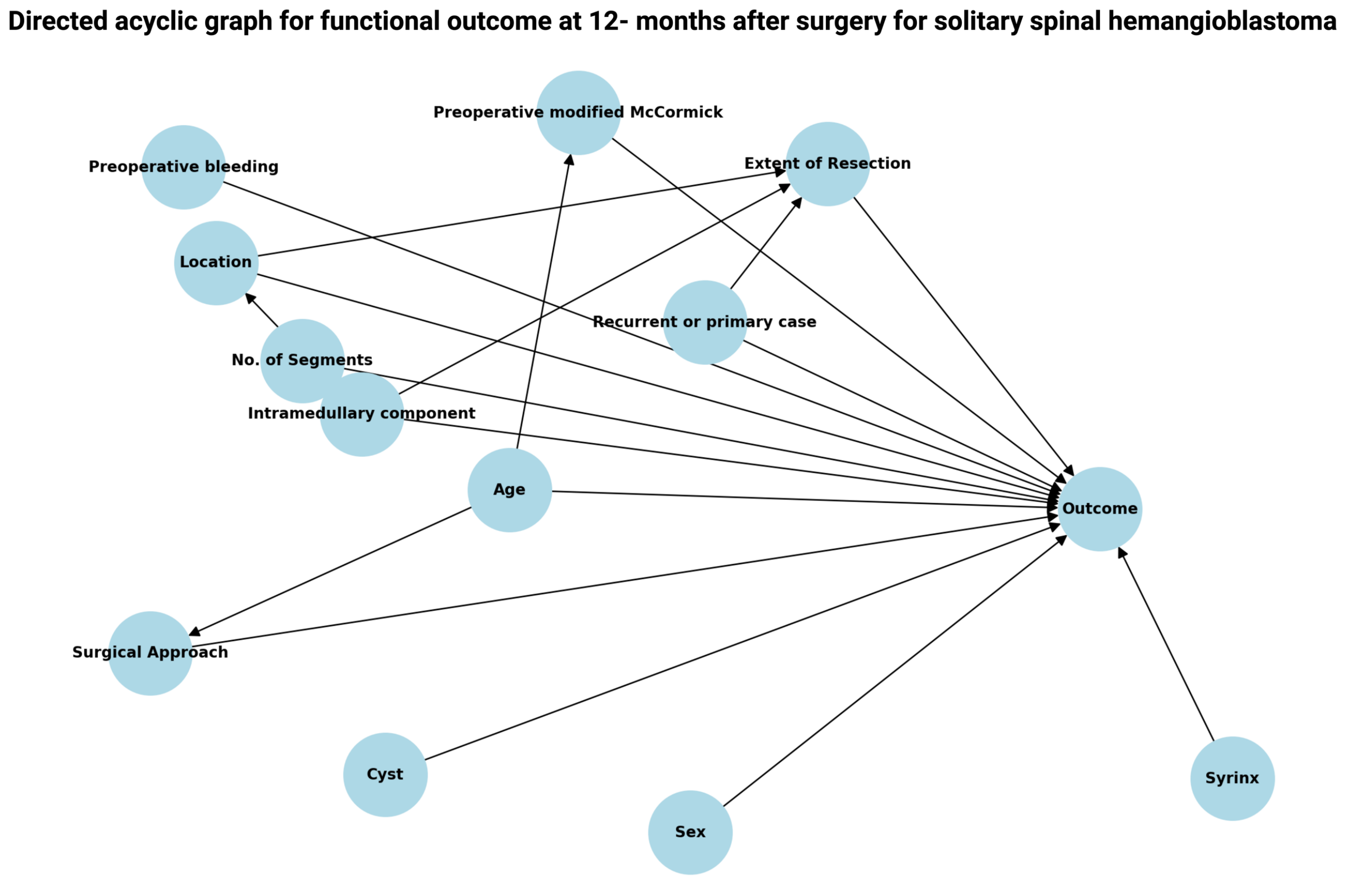

Supplement: noaf041_suppl_Supplementary_Materials [file noaf041_suppl_supplementary_materials.zip › supply/noaf041_suppl_Supplementary_Figure_S2.pdf]
